# Design of Curcumin and Flavonoid Derivatives with Acetylcholinesterase and Beta-Secretase Inhibitory Activities Using in Silico Approaches

**DOI:** 10.3390/molecules25163644

**Published:** 2020-08-10

**Authors:** Thai-Son Tran, Minh-Tri Le, Thanh-Dao Tran, The-Huan Tran, Khac-Minh Thai

**Affiliations:** 1 Department of Medicinal Chemistry, Faculty of Pharmacy, University of Medicine and Pharmacy at Ho Chi Minh City, Ho Chi Minh City 700000, Vietnam or tranthaison.hcmp@gmail.com (T.-S.T.); tranthanhdao@uphcm.edu.vn (T.-D.T.); 2 Department of Pharmaceutical Chemistry, Faculty of Pharmacy, College of Medicine and Pharmacy, Hue University, Hue City 530000, Vietnam; tthuan@hueuni.edu.vn; 3 School of Medicine, Vietnam National University Ho Chi Minh City, Ho Chi Minh City 700000, Vietnam

**Keywords:** acetylcholinesterase, beta-secretase, curcumin, flavonoid, in silico

## Abstract

Acetylcholinesterase (AChE) and beta-secretase (BACE-1) are the two crucial enzymes involved in the pathology of Alzheimer’s disease. The former is responsible for many defects in cholinergic signaling pathway and the latter is the primary enzyme in the biosynthesis of beta-amyloid as the main component of the amyloid plaques. These both abnormalities are found in the brains of Alzheimer’s patients. In this study, in silico models were developed, including 3D-pharmacophore, 2D-QSAR (two-dimensional quantitative structure-activity relationship), and molecular docking, to screen virtually a database of compounds for AChE and BACE-1 inhibitory activities. A combinatorial library containing more than 3 million structures of curcumin and flavonoid derivatives was generated and screened for drug-likeness and enzymatic inhibitory bioactivities against AChE and BACE-1 through the validated in silico models. A total of 47 substances (two curcumins and 45 flavonoids), with remarkable predicted pIC_50_ values against AChE and BACE-1 ranging from 4.24–5.11 (AChE) and 4.52–10.27 (BACE-1), were designed. The in vitro assays on AChE and BACE-1 were performed and confirmed the in silico results. The study indicated that, by using in silico methods, a series of curcumin and flavonoid structures were generated with promising predicted bioactivities. This would be a helpful foundation for the experimental investigations in the future. Designed compounds which were the most feasible for chemical synthesis could be potential candidates for further research and lead optimization.

## 1. Introduction

Alzheimer’s disease (AD), initially described by Alois Alzheimer in 1906 [1], is an irreversible neurodegenerative disorder with a high prevalence in the elderly [2]. It is estimated that there were about 50 million people suffering AD-related dementia globally in 2018, and this number will increase nearly three times in 2050 [3]. AD is creating a heavy socio-economic burden on the health care system of developed countries whose populations are aging [4]. The worldwide medical costs for dementia, including AD, were about 950 billion USD in 2015 and will be 2.5 trillion USD in 2030 and 9.1 trillion USD in 2050 [5].

On pathophysiology, the dominant hallmarks of AD are the presence of extracellular amyloid plaques and the intracellular neurofibrillary tangles with hyperphosphorylated Tau protein [6,7], loss of neural circuit integrity in the brain [8], and the alterations in synaptic/neuronal activities and neural metabolism [9]. There has been no effective treatment available for AD. The current AD therapies, acetylcholinesterase (AChE) inhibitors (rivastigmine, galantamine, donepezil) and *n*-methyl-D-aspartate receptor antagonist (memantine), solely slow down the progression of cognitive function decline, their therapeutic effects; thus, remain largely symptomatic, supportive and targeting late phases of the disease [6]. The expected effort is to discover disease-modifying therapies which can arrest or reverse the progression of the disease. The multifactorial nature of AD has shifted the paradigm of AD drug development from a single target to multiple targets, either with the multitarget-directed ligands approach or the cocktail therapy approach [10]. The drug targets that attracted much attention are acetylcholinesterase (AChE) and beta-secretase (BACE-1). These are two important enzymes in Alzheimer’s pathogenesis. AChE is responsible for the defects in the cholinergic signaling pathway [11], while BACE-1 is the main enzyme involved in biosynthesis of the key component of beta-amyloid plaques [12]. All of these disorders are present in the brains of Alzheimer’s patients.

Curcuminoids (curcumin and its analogs) and flavonoids are the most attractive groups of naturally derived compounds, with varieties of biological activities, and are proved to be beneficial against AD [13,14]. The therapeutic benefits of curcumin and flavonoid derivatives against AD occur via many pathways, including the activities on AChE [13,15] and BACE-1 [16,17].

The computational approaches, applied widely in the field of drug discovery, have successfully led to the development of many therapeutic agents [18,19,20]. With the use of these methods, the researcher could provide many valuable suggestions guiding the experimental phases of the drug development process. Recent studies showed that using computer-assisted drug design methods such as 2D-QSAR (two-dimensional quantitative structure-activity relationship), 3D-pharmacophore and molecular docking has been successful in discovering potential structures that inhibit AChE and BACE-1. This makes the prospects for finding new drugs in AD treatment more broad and promising [21,22,23,24].

**Figure 1:**
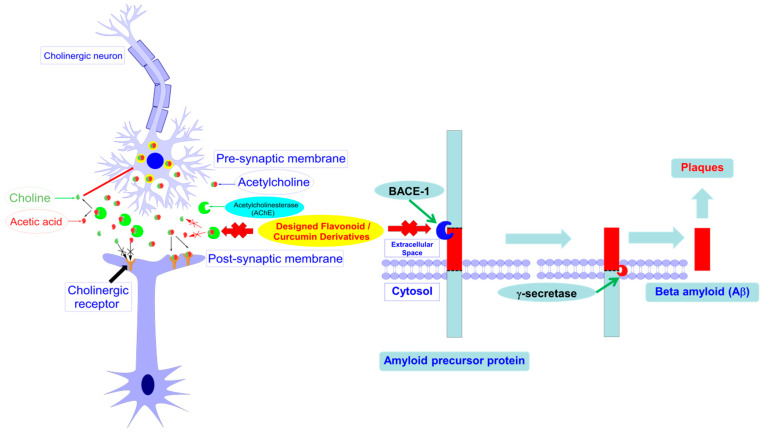
Designed curcumin and flavonoid derivatives with potential inhibitory activities against both acetylcholinesterase (AChE) and beta-secretase (BACE-1).

In this study, the in silico methods were employed for designing the curcumin and flavonoid derivatives with potential inhibitory activities against both AChE and BACE-1 (Figure 1) as an orientation for the chemical synthesis and experimental bioassays.

## 2. Results

### 2.1. Combinatorial Library of Curcumin and Flavonoid Derivatives

**Figure 2:**
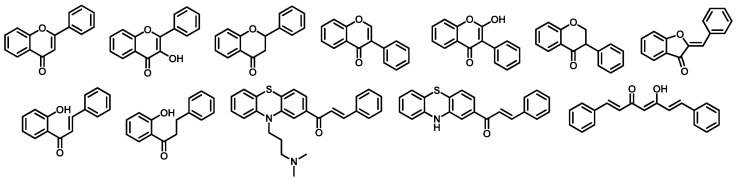
The scaffolds of curcumin and flavonoids used to design combinatorial library.

By using a combinatorial method with the scaffold structures of curcumin and flavonoids and substituents (R-groups) indicated in Figure 2, and Table 1, a library of 3,012,708 compounds (715,040 curcumins and 2,297,668 flavonoids) was designed. This library was then screened to find out compounds that are expected to have BACE-1 and AChE inhibitory activities.

### 2.2. 3D-Pharmacophore Models

**Figure 3:**
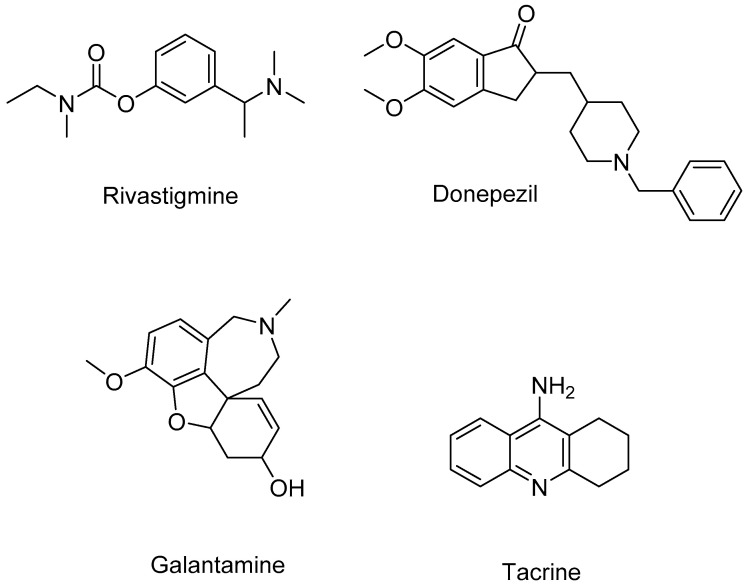
Structures of four compounds used for building pharmacophore models against AChE.

**Figure 4:**
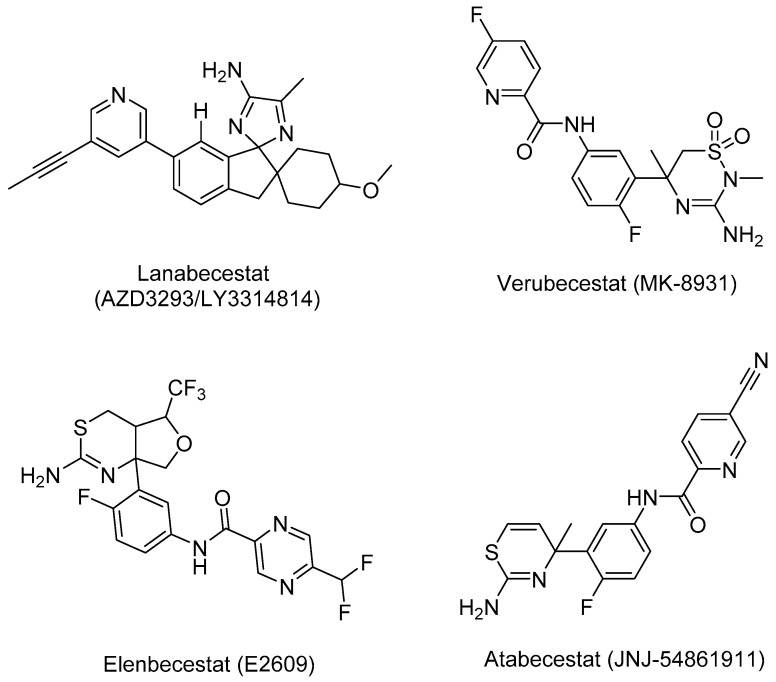
Structures of four compounds used for building pharmacophore models against BACE-1.

**Figure 5:**
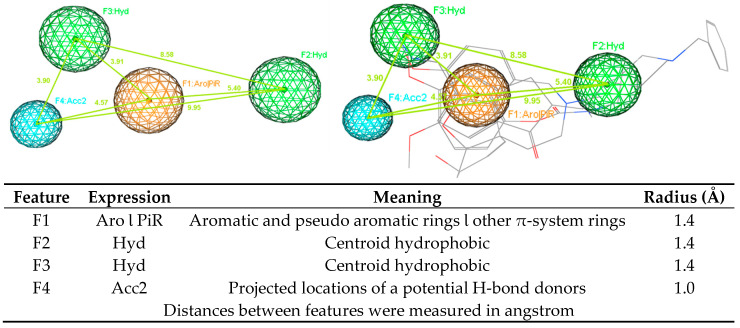
Pharmacophore model A1 (AChE).

**Figure 6:**
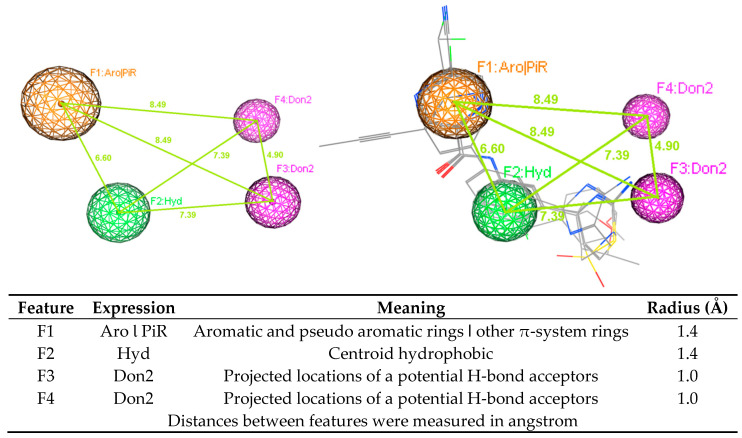
Pharmacophore model B1 (BACE-1).

Two pharmacophore model sets were developed, including 13 models (A1–A13) for AChE and 11 models (B1–B11) for BACE-1. These models were built with four clinical in used drugs or used to be approved substances (A1–A13), or four substances being in clinical development (B1–B11) (Figure 3 and Figure 4). These models were evaluated with the validation sets for their performance; from this the best models would be chosen for further process. In the validation process, the active and decoy sets were downloaded from the website http://dude.docking.org/. After processing the downloaded data sets, including removing duplicate entries based on Cluster codes tool in the software Molecular Operating Environment (MOE) 2008.10 [25], and eliminating the structures used in the training sets (if any), the obtained sets (Table 2) were used for validating built pharmacophore models. The evaluation results of two chosen models (A1 and B1) are indicated in Table 3, more detail about the validation results of developed pharmacophore models are showed in the Supplementary Materials (Tables S1 and S2). The validation results of developed pharmacophore models indicated that these models (Models A1 and B1 in Figure 5 and Figure 6, respectively) had the good performance and could be used in the virtual screening process with the high reliability.

### 2.3. Virtual Screening

Applying predictive models in the screening of designed combinatorial library, the results showed that from the initial library of more than 3 million substances, after the screening process, the number of potential substances obtained was 47 (two curcumins and 45 flavonoid). Specifically, after screened by Lipinski’s rule of five [27], the number of substances was reduced to 1,046,722 (6077 curcumins and 1,040,645 flavonoids). This number was then reduced to 4199 (two curcumins and 4197 flavonoids) after screening through two pharmacophore models. The data set of flavonoid derivatives was then refined the drug-likeness, the ability of crossing blood–brain barriers; and eliminated molecules containing substructures showing potent response in assays irrespective of the protein target, or to be putatively toxic, chemically reactive, metabolically unstable as well as to bear properties responsible for poor pharmacokinetics. After this refinement with the using of a free web tool SwissADME [28], the total remaining number of flavonoids was 45. These substances were predicted as the compounds that can cross the blood–brain barriers. They also do not violate any drug-like features, including Linpinski’s rule of five [27], Ghose filter [29], and the rules of Veber [30], Egan [31], or Muegge [32]. They were predicted as feasible synthetic accessibility (SA) with the scores of 2.1–3.76 (SA score ranges from 1 (very easy) to 10 (very difficult)) [28]. Two screened curcumin derivatives were predicted by SwissADME as the compounds that violate the Ghoser filter (with molecular weight >480, molecular refractivity >130, and the number of atoms >70). They were also predicted to have high GI (Gastrointestinal) absorption but not to cross the blood–brain barriers. These properties should be optimized in the further processes. The more detail of predicted properties of the screened compound are indicated in the Supplementary Materials.

**Figure 7:**
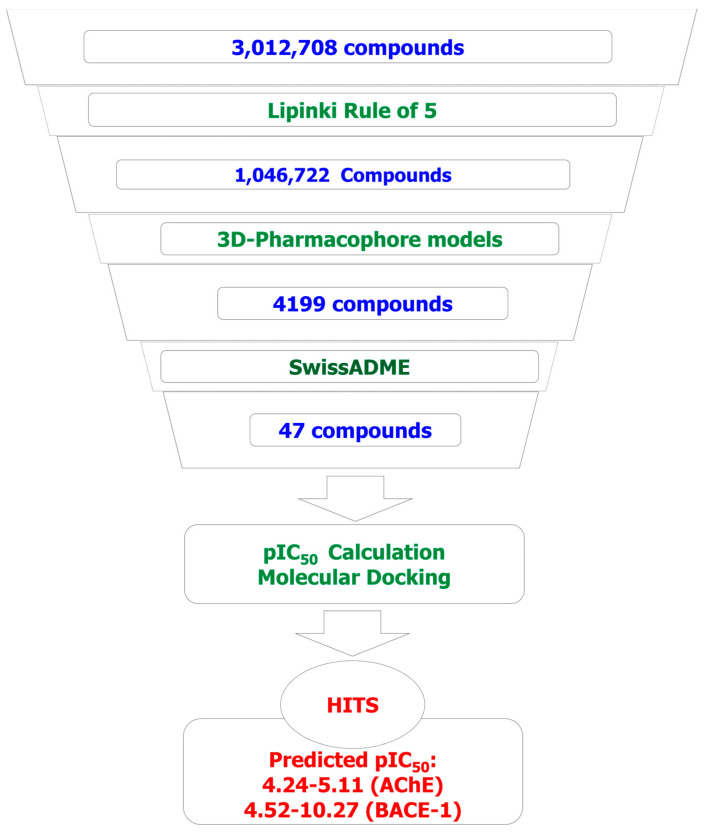
Virtual screening results.

All 47 compounds were then checked on Scifinder database [33] for the new structures with no record was retrieved. This could mean that all 47 designed substances are new in their structures. The predicted pIC_50_ values for these 47 screened derivatives (calculated using the 2D-QSAR models described below) range from 4.24–5.11 (AChE) and 4.52–10.27 (BACE-1). These compounds should be selected as potential candidates for synthesis and further evaluation. Virtual screening results and predicted bioactivities with docking scores of some of the most potential compounds are presented in Figure 7 and Table 6.

### 2.4. 2D-QSAR Models

**Figure 8:**
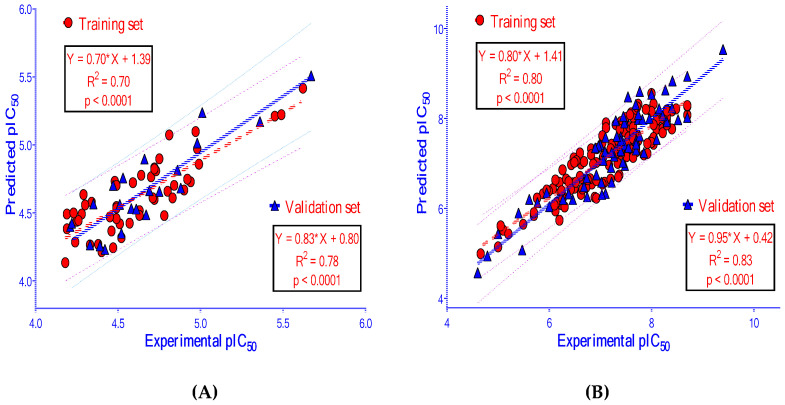
The correlation between experimental pIC_50_ (−logIC_50_) and predicted pIC_50_ from 2D-QSAR models built for (**A**) AChE and (**B**) BACE-1.

The results of building and validating 2D-QSAR models, presented in Table 4 and Figure 8, show that these models are satisfactory in the evaluation metrics with good predictability. These models could accurately predict the biological activity of new ligands. The datasets of compounds used in building 2D-QSAR models are provided in the Supplementary Materials (Tables S3 and S4). Chosen molecular descriptors used for building 2D-QSAR models are indicated in Table 5. A full list of descriptors calculated by the computational software is showed in the Supplementary Materials (Table S5).

### 2.5. Molecular Docking

**Figure 9:**
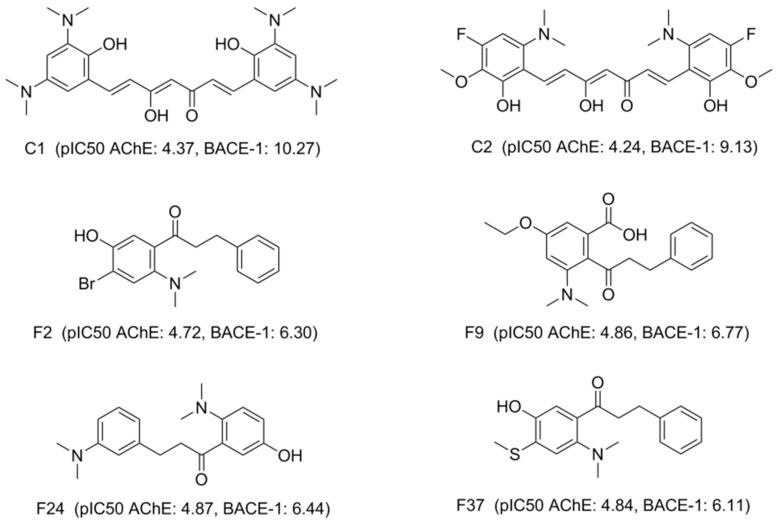
Structures of potential candidates with estimated bioactivities.

**Figure 10:**
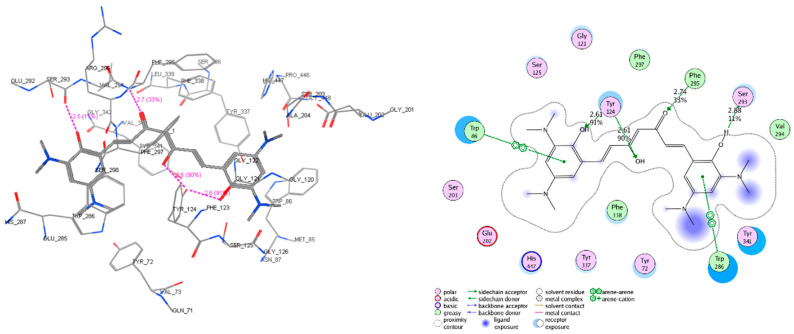
Interactive models of studied compound C1 with the residues in the binding pocket of AChE (code PDB 4EY7 – chain A). The residues, which have interactions with C1, include Tyr_72, Trp_86, Gly_121, Tyr_124, Ser_203, Ser_125, Trp_286, Glu_202, Ser_203, Ser_293, Val_294, Phe_295, Phe_297, Phe_338, Tyr_341, and His_447. In which, the hydrogen bonds with Tyr_124, Ser_293, Ph_295, and the arene−arene interactions are the most important.

**Figure 11:**
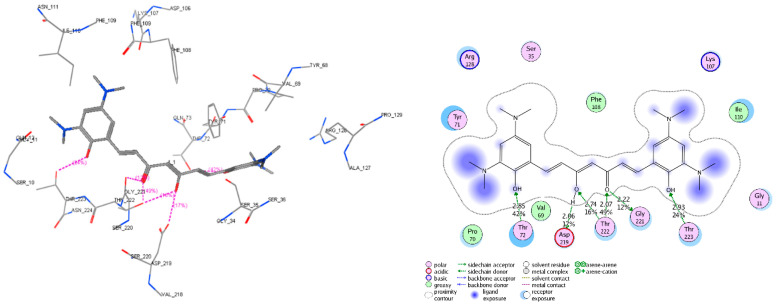
Interactive models of studied compound C1 with the residues in the binding pocket of BACE-1 (code PDB 3VEU). The residues, which have interactions with C1, include Gly_11, Ser_33, Val_69, Pro_70, Tyr_71, Thr_72, Lys_107, Phe_108, Ile_110, Arg_128, Gly_221, Thr_222, Thr_223, and Asp_219. In which, the hydrogen bonds with Thr_72, Asp_219, Thr_222, Gly_221, and Thr_223 are the most important.

The molecular docking models were built and validated as satisfactory, except for 5HTZ with all the RMSD (root mean square deviation) values were more than 2.0 (Å) (Table S6). Therefore, this co-crystalized complex was not used in the next docking process. The screened derivatives were then successfully docked, with some exceptions, into the binding cavity of AChE (1ACJ, 1DX6, 1EVE, 1W6R, 4EY6, 4EY7) and BACE-1 (3VEU, 4B78, 5HTZ, 5HU0, 5HU1). The substances with high predicted values of pIC_50_ and good docking scores (most negative) on both enzyme targets would be considered as the most potential candidates for chemical synthesis and biological activity testing. The docking results of several derivatives are presented in Table 6 and Figure 9, Figure 10 and Figure 11. The more detail of docking results predicted pIC_50_ of all screened compounds are shown in the Supplementary Materials with the structures of all 47 screened substances also indicated (Tables S7–S10).

### 2.6. AChE and β-Secretase Inhibitory Activities

The IC_50_ values for AChE and BACE-1 were performed for two synthesized compounds namely F9, F24, and summarized in Table 6. In vitro IC_50_ for AChE values of F9 and F24 were 30.05 ± 1.24 and 80.52 ± 3.07 µM, respectively. For BACE-1, the IC_50_ of 1.85 ± 0.33 and 3.52 ± 0.77 µM for F9 and F24, respectively, were determined. The results showed that the in vitro bioactivity is in consistent with in silico modelling with the pIC_50_ residues of 0.34–0.78 for AChE and 0.99–1.04 for BACE-1.

## 3. Discussion

In virtual screening, a library of drug-like substances can be used. However, these are commercial libraries, and the screening materials often do not have a new structure because they have been previously synthesized. Existing substances may also be restricted by patents on the method of use. In this study, QuaSAR-CombiGen tool was used to design a library of compounds for further explored. This utility could enumerate a virtual library of all possible products that are combinatorially generated from a set of fragment molecules. The virtual library is constructed by functionalizing central molecular fragments called scaffolds. The built-in library has the advantage of quickly creating a huge database of structures and avoiding data lost, as well as creating new structures. The construction of libraries with high chemical diversity is necessary when exploring new targets with very little known ligands, while targeted libraries based on the nature of known ligands may be also desired to identify hits with improved biological activity. Xing et al., [36] designed combinatorial libraries to search for novel soluble epoxide hydrolase (sEH) inhibitors based on a benzoxazole template forming conserved hydrogen bonds with the catalytic machinery of sEH. Consequently, screening of these libraries resulted to 90% hit rate and more than 300 submicromolar sEH inhibitors were finally discovered. In this study, a targeted library of compounds was also created based on the flavonoid and curcumin structures in an attempt to discover novel curcumin or flavonoid derivatives with improved biological effects on both enzyme, including AChE and BACE-1.

In this study, the predictive models of 2D-QSAR were built and validated with satisfactory in the evaluation metrics. A comparison of the statistical results obtained from the present QSAR models and previously published works is indicated in Table 7 and Table 8. Based on the statistical quality in the context of both internal and external validation criteria, the models reported in this study is statistically significant and robust enough to predict the biological activities of new ligands.

The 2D-QSAR model for AChE inhibitors was developed with the adjacency and distance matrix (BCUT_SlogP_3), physical property (*reactive*), partial charge (PEOE_VSA+1, PEOE_VSA−3), and subdivided surface areas (SlogP_VSA2, SMR_VSA2) descriptors. The 2D-QSAR model showed a positive correlation with BCUT_SLOGP_3, and a negative correlation with *reactive*, PEOE_VSA+1, PEOE_VSA−3, SlogP_VSA2, SMR_VSA2. It meant that new ligands with high BCUT_SLOGP_3, and low *reactive*, PEOE_VSA+1, PEOE_VSA−3, SlogP_VSA2, SMR_VSA2 values should have higher acetylcholinesterase inhibitory activities.

The 2D-QSAR model for BACE-1 inhibitors was developed with 11 molecular descriptors, including two adjacency and distance matrixes (*petitjean*, BCUT_PEOE_1), four atom counts and bond counts (*a_ICM, chiral_u, rings, a_Nn*), two partial charges (PEOE_VSA−0, PEOE_VSA−6), one physical properties (*logS*), and two subdivided surface areas (SlogP_VSA3, SlogP_VSA5). The 2D-QSAR model showed a positive correlation with the descriptors of *petitjean*, BCUT_PEOE_1, *a_ICM*, *rings*, *a_Nn*, PEOE_VSA−0, PEOE_VSA−6, SlogP_VSA3, SlogP_VSA5, and a negative correlation with *chiral_u*, *logS*. It means that new ligands with high *petitjean*, BCUT_PEOE_1, *a_ICM*, *rings*, *a_Nn*, PEOE_VSA−0, PEOE_VSA−6, SlogP_VSA3, SlogP_VSA5, and low *chiral_u***,** or *logS* values should have higher BACE-1 inhibitory activities.

Using ligand-based pharmacophore modeling to find novel acetylcholinesterase inhibitors and BACE-1 inhibitors is an approach employed in several studies [44,45,46,47]. From the developed models, novel inhibitors were discovered. In this study, the developed pharmacophore models were built for both AChE and BACE-1 inhibitors. Model A1 (AChE) had four features, including one point of aromatic and pseudo aromatic rings or other π-system rings, two points of centroid hydrophobic, and one point of projected locations for a potential H-bond donors. Model B1 (BACE-1) had also four features, including one point of aromatic and pseudo aromatic rings or other π-system rings, one point of centroid hydrophobic, and two points of projected locations for a potential H-bond donors. These two models shared three of the same types of features. These models were validated with the goodness-of-hit score (GH) of 0.62 (AChE) and 0.69 (BACE-1). With a good performance, these models could be used in the virtual screening process to discover potential structures with the dual activities on both enzymes. The virtual screening process was performed in a combination way, including the using of pharmacophore models, drug-likeness filtering and molecular docking. This combination is widely used in the search of novel biological activity substances from a database. Molecular docking was one step in the virtual screening process. This procedure also revealed the residues important for the functioning of AChE and BACE-1. The interactions of these residues with designed structures would explain for the potential of these substances to be the active candidates. From the screening process, 47 hits were obtained, in which there were 45 flavonoids and two curcumin derivatives. They were the ligands with new structures and were predicted to have the inhibitory activities against both AChE and BACE-1. In addition, 45 flavonoid ligands were predicted not to violate any drug-like characteristics. They were also considered blood−brain barriers permeants as well as easy for the synthesis. The two curcumin ligands were predicted as to not follow drug-likeness rules in some aspects. They were considered not to cross the blood−brain barriers but to have high GI absorption. These properties should be optimized in the further processes. Combining data from all these experiments will improve our knowledge about the ligand–AChE or BACE-1 interaction and enable the development of predictive classification and regression models. Although the tested set (F9 and F24) is by far too small to derive any hypothesis that validates our in silico modellings, this may be an initial hint that the residues are of 1.04 log10 value (10×) between the in silico and experimental data.

## 4. Materials and Methods

**Figure 12:**
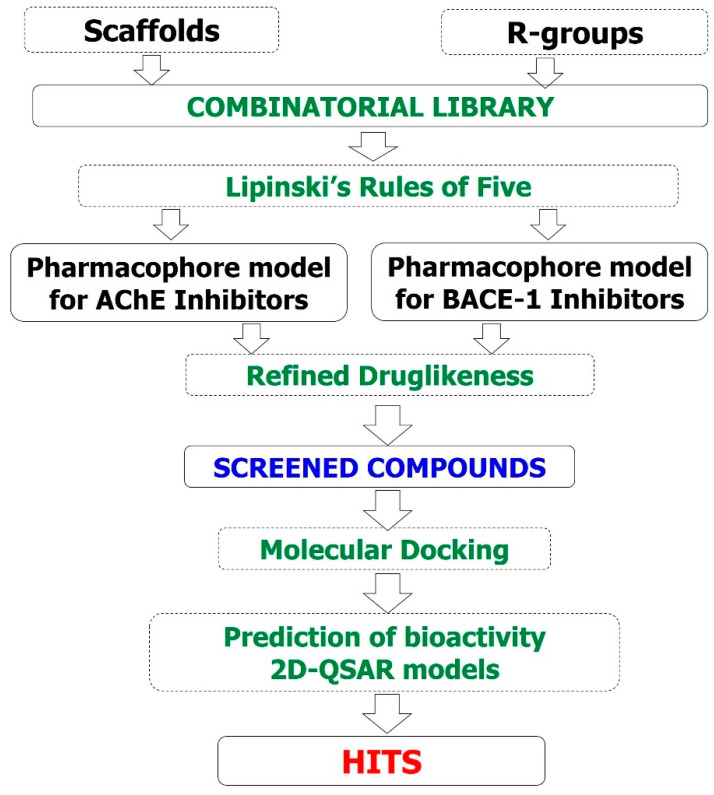
Study flowchart.

In silico models were built including 3D-pharmacophore, 2D-QSAR, and molecular docking. The models were used for virtually screening a combinatorial library of designed curcumin and flavonoid derivatives for AChE and BACE-1 inhibitory activities. All computation processes were performed on a computer system with the processor of Intel^®^ Core^TM^ i&-7700 CPU @ 3.60 Hz, 16.0 GB of RAM, the Visual Graphic Card of NVIDIA GeForce GT 1030 2GB, and the operating system of 64 bit Windows 10 (Microsoft, Redmond, WA, USA). The research process is summarized in Figure 12 and is described in detail as follows:

### 4.1. Design a Combinatorial Library of Curcumin and Flavonoid Compounds

The combinatorial library of curcumin and flavonoid compounds was designed using QuaSAR-Combigen tool in MOE 2008.10 software with the scaffolds and R-groups indicated in Figure 2 and Table 1. QuaSAR-CombiGen enumerated a virtual library of all possible products combinatorially generated by functionalizing the scaffolds. A combinatorial library is specified by a database of scaffold molecules, database of functional groups (R-groups), and connection information (attachment points) specifying where the R-groups attach on each scaffold (attachment points must be specified on both the R-group and the scaffold molecule). In this study, the scaffolds were specified the attachment points from one, two, to all carbon atoms of benzene rings. A single combinatorial product was constructed by attaching R-groups to a scaffold at marked attachment points, called *ports*. The entire combinatorial library was enumerated by exhaustively cycling through all combinations of R-groups at every attachment point on every scaffold. The virtual library was written to an output database and was then energy minimized to obtain a lower energy conformation for each molecule.

### 4.2. Building and Validating Pharmacophore Models

Pharmacophore models were built in MOE 2008.10 using the *Pharmacophore Elucidation* application. Conformations of the compounds were generated and used to create queries with good coverage levels in almost all molecular compounds in the training set. The *Active Coverage,* which specifies the number of molecules that an obtained query must match in order for it to be considered further and outputted, employed in this study was 0.8. *Feature Limit* was in the default value of 5, this is the maximum number of features that an output pharmacophore was permitted to contain. *Query Cluster* parameter was set to default value of 1.25 Å. This number specified an RMSD value used to cluster the queries prior to overlapping and scoring. All obtained models were then validated to evaluate their performance. Enrichment factor (EF) and goodness-of-hit score (GH) were calculated. The GH score ranges from 0, which indicates the null model, to 1, which indicates the ideal model. When GH score is 0.6–0.8, the model is considered to have a good performance [26].

### 4.3. Building 2D-QSAR Models

#### 4.3.1. Data Collection

The BACE-1 inhibitor database was collected from scientific papers [48,49,50,51,52,53,54,55,56,57,58,59], and AChE inhibitors were curated from the CheMBL database [60]. After processing input data, including removing substances with similar structural characteristics (based on the Cluster codes tool in MOE 2008.10), filtering substances with the same biological test method, and IC_50_ values correction to get 215 BACE-1 inhibitors with the same FRET (fluorescence resonance energy transfer) test method, and 72 AChE inhibitors with the same Ellman’s test method on AChE of the *Electrophorus electricus* with galantamine as the reference substance. The IC_50_ values of the substances were then converted to pIC_50_ to facilitate the calculation. Structures of compounds in the databases were built and energy minimized in MOE 2008.10 with the default setting. The final obtained databases were then used to build 2D-QSAR models with optimal molecular descriptors.

#### 4.3.2. Molecular Descriptors Calculation and Processing

2D molecular descriptors were calculated using MOE 2008.10 software, and then processed to eliminate redundant or unrelated ones to increase the quality of predictive models, while reducing calculating time [61]. Firstly, RapidMiner 5.3.013 [62] was used to eliminate useless descriptors (ones with fixed values for at least 80% of the total substances). The descriptors correlated with each other (with correlation coefficient >0.9) were also deleted. The software Weka 3.8 [63] was then used to select optimal descriptors for the predictive models. The method used in this stage was *BestFirst* and the attribute selection mode was *Use full training set.*

#### 4.3.3. Building and Validating of 2D-QSAR Models

Each database of BACE-1 and AChE inhibitors was divided into training set and validation set in a ratio of 80% to 20% using *Diverse Subset* and *Rand* methods in MOE 2008.10. The *Diverse Subset* utility was used to rank entries in a database based on their distance from each other. While the *Rand* function was used to split randomly the database of compounds, each of which was assigned a random number between 0 and 1. 2D-QSAR models were built using *Partial Least Square* (PLS) regression. This is the most straightforward quantitative multivariate modelling method, which models the relationship between two data matrices, X (independent variables, descriptors) and Y (target variable, bioactivity). The developed linear regression model can predict the quantitative response values from the linear function of molecular variables. It offers an advantages such as can be useful in the analysis of data with strongly collinear, noisy and several X variables as well as simultaneous modelling of several target variables Y [64].

The models then validated by the values RMSE (root-mean-square error), R^2^ (squared correlation coefficient), RMSE_LOO_ (cross-validated root-mean-square error), R^2^_LOO_ (cross-validated squared correlation coefficient), and more widely used metrics $r_{m}^{2}$, ${r’}_{m}^{2}$, $\bar{r_{m}^{2}}$, $∆r_{m}^{2}$; R^2^_PRED_, or CCC (concordance correlation coefficient) [65], and $Q_{F3}^{2}$.

These parameters were calculated according to the Equations (1)–(11).

(1) $$\mathrm{RMSE}=\sqrt{\frac{\sum_{i=1}^{n_{TR}}{\left({\hat{y}}_{i}-y_{i}\right)}^{2}}{n}}$$

(2) $$R^{2}=1-\frac{\;\sum_{i=1}^{n_{TR}}{\left({\hat{y}}_{i}-y_{i}\right)}^{2}}{\sum_{i=1}^{n_{TR}}{\left(y_{i}-{\bar{\;y}}_{i}\right)}^{2}}$$

(3) $${\mathrm{RMSE}}_{\mathrm{LOO}}=\sqrt{\frac{\sum_{i=1}^{n_{TR}}{\left(\hat{y}{’}_{i}-y_{i}\right)}^{2}}{n}}$$

(4) $${R^{2}}_{\mathrm{LOO}}=1-\frac{\sum_{i=1}^{n_{TR}}{\left(\hat{y}{’}_{i}-y_{i}\right)}^{2}}{\sum_{i=1}^{n_{TR}}{\left(y_{i}-{\bar{\;y}}_{i}\right)}^{2}}$$

(5) $${R^{2}}_{\mathrm{PRED}}=1-\frac{\sum_{I=1}^{n_{VAL}}{\left(y_{pred\left(validation\right)}-y_{validation}\right)}^{2}}{\sum_{I=1}^{n_{VAL}}{\left(y_{validation}-{\bar{y}}_{training}\right)}^{2}}$$

(6) $$r_{m}^{2}=r^{2}\left(1-\sqrt{r^{2}-r_{0}^{2}}\right)$$

(7) $$r{’}_{m}^{2}=r{’}^{2}\;\left(1-\sqrt{{r^{\prime }}^{2}-{r^{\prime }}_{0}^{2}}\right).\;$$

(8) $$\bar{r_{m}^{2}}=\frac{r_{m}^{2}+r{’}_{m}^{2}}{2}$$

(9) $$∆r_{m}^{2}=\left|r_{m}^{2}-r{’}_{m}^{2}\right|$$

(10) $$CCC=\frac{2\sum_{i=1}^{n_{VAL}}\left(y_{i}-\bar{y}\right)({\hat{y}}_{i}-\bar{\hat{y}})}{\sum_{i=1}^{n_{VAL}}{\left(y_{i}-\bar{y}\right)}^{2}+\sum_{i=1}^{n_{VAL}}{\left(\hat{y_{i}}-\bar{\hat{y}}\right)}^{2}+n_{VAL}{\left(\bar{y}-\bar{\hat{y}}\right)}^{2}}$$

(11) $$Q_{F3}^{2}=1-\frac{\sum_{i=1}^{n_{VAL}}{\left(y_{i}-{\hat{y}}_{i}\right)}^{2}/n_{VAL}}{\sum_{i=1}^{n_{TR}}{\left(y_{i}-{\bar{y}}_{training}\right)}^{2}/n_{TR}\;}$$

In Equations (1), (2), (10), and (11), $y_{i}$ and ${\hat{y}}_{i}$ are, respectively, the observed and predicted activity values; while $\bar{y}$ and $\bar{\hat{y}}$ are, respectively, the mean values of $y_{i}$ and ${\hat{y}}_{i}$. In Equations (3) and (4),$\;y_{i}$ and $\hat{y}{’}_{i}$ are, respectively, the observed and predicted activity values in LOO cross-validation. Equations (6) and (7) were utilized to calculate correlation coefficients between observed and predicted activity values of the compounds of validation set with ($r^{2}$) or without intercept ($r_{0}^{2}$) in case of using predicted data on the y-axis and experimental data on the x-axis, while ($r{’}^{2}$) and (${r^{\prime }}_{0}^{2}$) are, respectively, the same coefficients in the opposite case. The most stringent validation criteria thresholds including $r_{m}^{2}$ ≥ 0.65; CCC ≥ 0.85; $\bar{r_{m}^{2}}$ ≥ 0.5; $∆r_{m}^{2}$ ≤ 0.2; and $Q_{F3}^{2}$ > 0.6 were applied to verify the external predictivity of good models [66,67,68,69].

### 4.4. Molecular Docking Procedure

#### 4.4.1. Ligand Preparation

Ligand molecules were prepared directly in Sybyl X 2.0 [70]. In the energy minimization process, the *Conj Grad* method was chosen and the structures of molecules were optimized until the minimum energy change ≤0.0001 kcal.mol^−1^. *Gasteiger−Huckel* charges were assigned to the structure atoms and the maximum number of iterations to perform during minimization was set to 10,000. Molecular dynamic process was proceeded to obtain conformations with the minimum global energy. The method used in this step was *Simulated Annealing*. In this method, the molecules were heated at 700 °K in a period of 1000 femtoseconds, then they were cooled down to 200 °K in another period of 1000 femtoseconds to approach the stable states from which their final conformations were obtained. The process runs in five cycles to figure out different necessary structures. Finally, the energy minimization process was performed one more time and the steric energies of final conformations were specified.

#### 4.4.2. Docking and Results Evaluation

Docking protocols were validated by the method of pose selection [71]. In this method, docking was carried out initially with co-crystallized structure to validate the protocol. The co-crystallized ligand was re-docked within the binding pocket of BACE-1 and AChE, and the RMSD value between the best re-docked conformation and the native one was calculated. If the RMSD is ≤2.0 Å, the used docking protocol could be considered as validated [72]. In this study, the co-crystallized complexes employed for AChE were 1ACJ, 1DX6, 1EVE, 1W6R, 4EY6, 4EY7 and for BACE-1 were 3VEU, 4B78, 5HTZ, 5HU0, 5HU1. These were the complexes with high resolution and the co-crystallized ligands were the drugs used in clinical or in clinical development. With this selection, the probability of docked compounds to be reached further optimization would be high. The protein complexes were downloaded from protein data bank [73] and prepared in MOE 2008.10 using LigX tool. This tool helped to detect and visualize the ligand in the binding site. Docking process was done using FlexX program in BioSolveIT LeadIt 2.0.2 [74]. This program applied the flexible-based docking methodology with an incremental construction algorithm to search the ligand conformations, and empirical scoring functions to score and rank the docking poses [75,76]. In this study, the docking process was done with following options: The binding sites of proteins were identified based on co-crystallized ligands and the presence of important residues (within a default radius of 6.5 Å). Unbound water molecules were eliminated. *Triangle Matching* algorithm was used to place the base fragment; the maximum number of solutions per iteration was 1000, the maximum number of solution per fragmentation was 200, and the number of best poses of each molecular compound in binding complex to retain for analyzing interaction was 10. These poses were scored and ranked ascending. The score was the predicted binding free energy between the ligand and its target. Interactions between molecular compounds with the active sites of the enzymes, such as hydrogen bonds, and *van der Waals* (detected by the exposure of hydrophilic and hydrophobic surface with molecular compounds and binding points), π−π, cation−π, ionic interactions were depicted and analyzed by MOE 2008.10.

### 4.5. Chemistry

**Figure 13:**
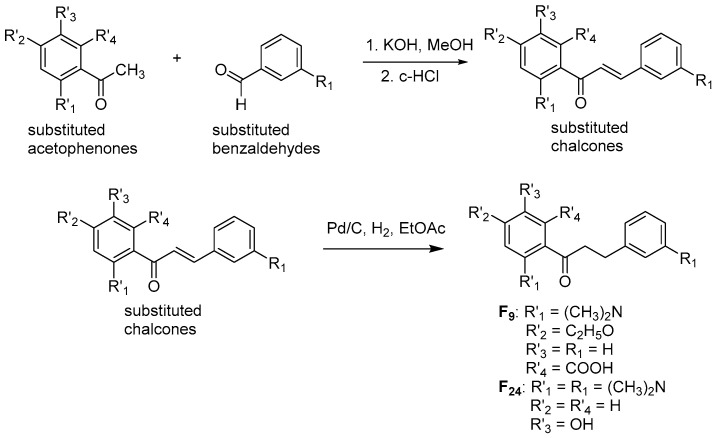
Chemical synthesis of two flavonoid derivatives **F9** and **F24**.

Two dihydrochalcones F9 and F24 were synthesized according to the reactions indicated in Figure 13. Firstly, substituted chalcone derivatives were prepared by a Claisen−Schmidt condensation reaction [77] of substituted acetophenones and aldehydes in equimolar quantities with KOH at room temperature. Chalcone derivatives then underwent hydrogenation in a H_2_ atmosphere with 10% a carbene complex of palladium (Pd−C) as catalyst and ethyl acetate (EtOAc) as solvent for 24–48 h [78] to obtain F9 and F24, whose structures were elucidated by ^1^H-NMR and ^13^C-NMR spectra (indicated below and in the Supplementary Materials).

*3-(dimethylamino)-5-ethoxy-2-(3-phenylpropanoyl)benzoic acid (F9)*: ^1^H-NMR (500 MHz, DMSO-*d6*) δ (ppm): 12.83 (s, 1H), 7.24 (m, 5H), 7.12 (d, 1H), 6.64 (d, 1H), 4.08 (q, *J* = 7 Hz, 2H), 3.25 (t, *J* = 6 Hz, 2H), 2.94 (s, 6H), 2.87 (m, 2H), 1.38 (t, *J* = 7.0 Hz, 3H). ^13^C-NMR (125 MHz, DMSO-*d6*) δ 202.04, 169.97, 163.06, 154.09, 141.59, 130.21, 128.51, 128.49, 126.30, 117.91, 107.34, 103.17, 63.61, 43.63, 43.43, 30.66, 14.41.

*1-(2-(dimethylamino)-5-hydroxyphenyl)-3-(3-(dimethylamino)phenyl)propan-1-one (F24)*: ^1^H-NMR (500 MHz, DMSO-*d6*) δ (ppm): 9.05 (s, 1H), 7.11 (m, 1H), 7.08 (dd, 1H), 6.94 (dd, 1H), 6.91 (ddd, 1H), 6.81 (m, 1H), 6.74 (m, 2H), 3.26 (t, *J* = 7.4, 2H), 3.00 (m, 3H), 2.93 (s, 3H), 2.76 (s, 6H), 2.73 (t, *J* = 7.4, 2H). ^13^C-NMR (125 MHz, DMSO-*d6*) δ 203.36, 152.35, 151.78, 145.49, 140.58, 129.25, 127.62, 122.85, 119.38, 116.97, 115.93, 114.96, 112.25, 44.89, 40.72, 40.42, 30.35.

### 4.6. AChE Inhibition Assay

AChE-inhibitory activities of F9, F24 were determined by the Ellman’s method [67], using galantamine as a reference compound. Acetylcholinesterase (AChE, E.C. 3.1.1.7, from electric eel), 5,5′-dithiobis-(2-nitrobenzoic acid) (DTNB), acetyl-thiocholine iodide (ATCI), galantamine were purchased from Sigma Aldrich (St. Louis, MO, USA). Tested compounds were dissolved in a minimum volume of 10% methanol in Tris buffer pH 8 to provide a final concentration range: 120 μM; 60 μM; 30 μM; 15 μM; 7.5 μM. All samples were assayed in triplicate, and bioactivity was reported with SEM. The method was as described earlier [79].

### 4.7. β-Secretase Inhibition Assay

β-secretase (BACE-1) Activity Detection Kit (Fluorescent) was purchased from Sigma-Aldrich and used to determine the effect of the F9, F24 on β-secretase activity. The assay was carried out according to the manufacturer’s protocol. The enzyme solution (0.3 units/μL, 2 μL) was reacted with the 50 μM of the substrate (7-methoxycumarin-4-acetyl-(Asn_670, Lue_671)-amyloid β/A4 precursor protein 770 fragment 667-676-(2,4-dinitrophenyl))Lys-Arg-Arg amide trifluoroacetate salt and sulfated polysaccharide samples (2–5 mg/mL) in a fluorescence assay buffer in different wells. Baseline readings were measure immediately on a Fluorescence Spectrophotometer Hitachi F-7000 (excitation: 320 nm; emission: 405 nm) and repeated after 2 h incubation at 37 °C. All samples were assayed in triplicate, and bioactivity was reported with SEM. The method was as described earlier [80,81].

## 5. Conclusions

Computer-assisted drug design has the advantage of greatly reducing research time in search of new biologically active compounds. This study carried out a systematic sequential research method, including building a library of curcumin and flavonoid derivatives designed on computers with different substituents (R-groups) on different structural scaffolds, developing models for virtual screening and biological activity prediction. The built-in library has the advantage of quickly creating a huge database of structures and avoiding data lost, as well as creating new structures. The models built from this study were evaluated and met the validating criteria for each one, proving that these were reliable models for predicting the biological activities of new structures. This study carried out a combination of ligand-based drug design (2D-QSAR, 3D-pharmacophore) and structure-based (docking) method. This combination helped to comprehensively assess the effects of molecular descriptors in 2D (QSAR) and 3D (pharmacophore, docking), as well as factors that belong to both the target and studied ligands, to biological activity. This combination could also help to gain the reliability of predictive models. Through virtual screening and pIC_50_ value prediction, the potential candidates, with new structures, had good predictive activities; in which, two curcumin derivatives were the most potent for BACE-1. All 45 designed flavonoids were also predicted to have many features of drugs and feasible synthetic accessibilities. The in vitro assays on AChE and BACE-1 were performed for two compounds and confirmed the in silico results with the maximum difference 10 times. Therefore, the results of this study could be considered as valuable suggestions for further experimental researches and directions.

## Figures and Tables

**Table 1:** R-groups used to design combinatorial library of curcumin and flavonoid derivatives.

-OH	-OCH_3_	-OC_2_H_5_	-OCOCH_3_
-F	-Cl	-Br	-I
-NO_2_	-NH_2_	-N(CH_3_)_2_	-NHCOCH_3_
-CH_2_CHH=CH_2_	-COOH	-COOCH_3_	-COOC_2_H_5_
-CN	-CONH_2_	-SO_2_NH_2_	-SH
-SCH_3_	-SC_2_H_5_	C_6_H_5_CH_2_O-	

**Table 2:** Databases for building and validation of the pharmacophore models.

AChE			BACE-1		
Training Set	Validation Sets		Training Set	Validation Sets	
	Active	Decoy		Active	Decoy
04	655	26369	04	436	18217

**Table 3:** Pharmacophore model validation by goodness-of-hit (GH) score method.

No.	Parameter	Pharmacophore Model	
		A1	B1
1	Total molecules in database *(D)*	27024	18653
2	Total number of actives in database *(A)*	655	436
3	Total hits (*Ht*)	914	438
4	Active Hits (*Ha*)	524	305
5	%Yield of actives [(*Ha/Ht*) × 100]	57.33	69.63
6	%Ratio of actives [(*Ha/A*) × 100]	80	69.95
7	Enrichment factor (*E*), [(*Ha* × *D*)/(*Ht* × *A*)]	23.65	29.79
8	False negatives [*A* – *Ha*]	131	131
9	False positives [*Ht* − *Ha*]	390	133
10	Goodness-of-hit score (GH*)	0.62	0.69

[(Ha/4HtA)(3A + Ht) *×* (1 − (Ht − Ha)/(D − A))]; GH score of 0.6–0.8 indicates a very good model [26].

**Table 4:** Two-dimensional quantitative structure-activity relationship models (2D-QSAR) of the inhibitors against acetylcholinesterase (AChE) and beta-secretase (BACE-1).

AChE														
***Model AT***							***Model AF: Full data set (n = 72)***							
pIC_50_ = −0.92791							pIC_50_ = −1.00890							
+2.34847×BCUT_SLOGP_3							+2.38027×BCUT_SLOGP_3							
−0.14990×reactive							−0.11002×reactive							
−0.00355×PEOE_VSA+1							−0.00391×PEOE_VSA+1							
−0.00514×PEOE_VSA-3							−0.00480×PEOE_VSA-3							
−0.00219×SlogP_VSA2							−0.00202×SlogP_VSA2							
−0.00447×SMR_VSA2							−0.00387×SMR_VSA2							
**Internal Validation**					**External Validation**									
*N*	RMSE	R^2^	RMSE_LOO_	R^2^_LOO_	*n*	RMSE		R^2^	R^2^_(PRED)_	$r_{m}^{2}$	$\bar{r_{m}^{2}}$	$∆r_{m}^{2}$	CCC	$Q_{F3}^{2}$
50	0.18	0.70	0.22	0.57	22	0.16		0.78	0.78	0.64	0.69	0.11	0.88	0.72
**BACE-1**														
***Model BT***							***Model BF: Full Data Set (N = 215)***							
pIC_50_ = 1.26826							pIC_50_ = 1.01351							
+0.87076×petitjean							+0.59775×petitjean							
+6.37086×BCUT_PEOE_1							+4.85517×BCUT_PEOE_1							
+3.30481×a_ICM							+3.13351×a_ICM							
−0.47753×chiral_u							−0.50839×chiral_u							
+0.08513×rings							+0.02540×rings							
+0.15746×a_nN							+0.16067×a_nN							
+0.00608×PEOE_VSA-0							+0.00577×PEOE_VSA-0							
+0.02183×PEOE_VSA-6							+0.01771×PEOE_VSA-6							
−0.25952×logS							−0.26227×logS							
+0.00893×SlogP_VSA3							+0.00920×SlogP_VSA3							
+0.00944×SlogP_VSA5							+0.01101×SlogP_VSA5							
**Internal Validation**					**External Validation**									
*N*	RMSE	R^2^	RMSE_LOO_	R^2^_LOO_	*n*	RMSE		R^2^	R^2^_(PRED)_	$r_{m}^{2}$	$\bar{r_{m}^{2}}$	$∆r_{m}^{2}$	CCC	$Q_{F3}^{2}$
150	0.37	0.80	0.40	0.77	65	0.41		0.83	0.81	0.79	0.76	0.05	0.91	0.76

RMSE (root-mean-square error), R^2^ (squared correlation coefficient), RMSE_LOO_ (cross-validated root-mean-square error), R^2^_LOO_ (cross-validated squared correlation coefficient), CCC (concordance correlation coefficient).

**Table 5:** Chosen descriptors used for building 2D-QSAR models.

Code	Category	Description
BCUT_SlogP_3	Adjacency and distance matrix	A Burden’s parameter using atomic contribution to logP (using the Wildman and Crippen SlogP method [34]) instead of partial charge.
BCUT_PEOE_1	Adjacency and distance matrix	A descriptor relating topological shape and partial charges.
petitjean	Adjacency and distance matrix	Value of (diameter-radius)/diameter.
reactive	Physical property	An indicator of the presence of reactive groups. A non-zero value indicates that the molecule contains a reactive group. The table of reactive groups is based on the Oprea set [35] and includes metals, phospho-, N/O/S-N/O/S single bonds, thiols, acyl halides, Michael Acceptors, azides, esters, etc.
logS	Physical property	The log of the aqueous solubility (mol/L).
PEOE_VSA-0, PEOE_VSA+1, PEOE_VSA-3, PEOE_VSA-6	Partial charge	Sum of the proximate accessible *van der Waals* surface area (Å^2^), *v_i_,* calculation for each atom over all the atoms *i*, such that partial charge of atom *i* is in a specified range.
SlogP_VSA2, SlogP_VSA3, SlogP_VSA5	Subdivided surface areas	Sum of the proximate accessible *van der Waals* surface area (Å^2^), *v_i_*, calculated for each atom over all the atoms, such that partition coefficient for atom *i* is in a specified range.
SMR_VSA2	Subdivided surface areas	Sum of the proximate accessible *van der Waals* surface area (Å^2^), *v_i_*, calculation for each atom over all the atoms *i*, such that molar refractivity for atom *i* is in a specified range.
a_ICM	Atom counts and bond counts	The entropy of the element distribution in the molecule (including implicit hydrogens but not lone pair pseudo-atoms).
chiral_u	Atom counts and bond counts	The number of unconstrained chiral centers.
rings	Atom counts and bond counts	The number of rings.
a_Nn	Atom counts and bond counts	The number of nitrogen atoms.

**Table 6:** Predicted bioactivities and docking scores of some potential candidates.

AChE											
Ligand	Predicted pIC_50_	In Vitro IC_50_ (µM)	In Vitro pIC_50_	Docking Score (kJ.mol^−1^)							
				1ACJ	1DX6	1EVE	1W6R	4EY6 (chain A)	4EY6 (chain B)	4EY7 (chain A)	4EY7 (chain B)
C1	4.37	-	-	Not docked	−24.13	−25.62	−25.47	Not docked	Not docked	−34.38	−36.23
C2	4.24	-	-	Not docked	−30.97	−23.97	−25.19	−23.21	−12.72	−23.56	−31.90
F2	4.72	-	-	−21.38	−21.18	−23.30	-27.09	−21.95	−21.56	−28.70	−30.99
**F9**	4.86	30.05 ± 1.24	4.52 ± 0.02	−20.27	−28.46	−25.53	−25.80	−25.63	−27.26	−25.17	−27.11
**F24**	4.87	80.52 ± 3.07	4.09 ± 0.02	−20.19	−21.08	−20.87	−21.53	−24.10	−23.66	−25.27	−26.59
F37	4.84	-	-	−20.58	−22.46	−21.81	−22.98	−21.02	−22.75	−23.34	−22.45
**BACE-1**											
				3VEU	4B78	5HU0 (chain A)	5HU0 (chain B)	5HU1 (chain A)	5HU1 (chain B)		
C1	10.27	-	-	−24.28	−10.23	−17.28	−22.09	−17.39	−14.74		
C2	9.13	-	-	−24.04	−24.64	−27.00	−16.51	−25.78	−17.79		
F2	6.30	-	-	−19.51	−13.11	−14.79	−14.92	−16.47	−17.20		
**F9**	6.77	1.85 ± 0.33	5.73 ± 0.08	−21.34	−15.98	−18.53	−17.83	−20.32	−19.49		
**F24**	6.44	3.52 ± 0.77	5.45 ± 0.10	−22.39	−14.36	−18.58	−17.07	−15.09	−16.19		
F37	6.11	-	-	−21.87	−13.66	−17.39	−15.80	−16.61	−15.09		

**Table 7:** Comparison of this study with previous published works on AChE.

*Source*	*Model*	*Training Set*			*Validation Set*	
		*N*	*R^2^*	*Q^2^*	*n*	*R^2^_PRED_*
**This study**	**PLS**	**55**	**0.70**	**0.57**	**22**	**0.78**
Roy et al. 2018 [37]	MLR	284	0.52–0.74	0.50–0.71	142	0.50–0.63
Niraj et al. 2015 [38]	PLS	24	0.78	0.70	11	0.66
Abuhamdah et al. 2013 [24]	GFA−MLR	68	0.94	0.92	17	0.84
Solomon et al. [39]	GFA	53	0.86	0.80	26	0.86

PLS: Partial least squares; MLR: Multiple linear regression; GFA: Genetic function approximation.

**Table 8:** Comparison of this study with previous published works on BACE-1.

*Source*	*Model*	*Training Set*			*Validation Set*	
		*N*	*R^2^*	*Q^2^*	*n*	*R^2^_PRED_*
**This study**	**PLS**	**150**	**0.80**	**0.77**	**65**	**0.81**
Kumar et al. 2019 [21]	PLS	76	0.83	0.80	22	0.85
Ambure et al. 2016 [40]	PLS	52	0.83	0.76	22	0.81
Ambure et al. 2016 [40]	MLR	51	0.83	0.76	22	0.80
Jain et al. 2013 [41]	MLR	20	0.90	0.90	7	0.90
Hossain et al. 2013 [42]	CoMFA	71	1.00	0.77	35	0.77
Hossain et al. 2013 [42]	CoMSIA	71	1.00	0.73	35	0.71
Hossain et al. 2013 [42]	PLS	71	0.94	0.79	35	0.71
Chakraborty et al. 2017 [43]	LHM	20	0.94	0.91	10	0.86
Roy et al. 2018 [37]	MLR	51	0.76–0.83	0.71–0.76	23	0.75–0.91

PLS: Partial least squares; MLR: Multiple linear regression; CoMFA: Comparative molecular field analysis; CoMSIA: Comparative similarity indices analysis; LHM: Linear heuristic method.

## Supplementary Materials

The following are available online, Table S1: Pharmacophore model validation by goodness-of-hit score (GH) score method (AChE), Table S2: Pharmacophore model validation by goodness-of-hit score (GH) score method (BACE-1), Table S3: Dataset of 72 compounds used in the building of 2D-QSAR model for AChE inhibitors, Table S4: Dataset of 215 compounds used in the building of 2D-QSAR model for BACE-1 inhibitors, Table S5: List of 2D molecular descriptors computed using MOE 2008.10 software, Table S6: Re-docking results (RMSD values in Å), Table S7: Results of molecular docking of curcumins, Table S8: Results of molecular docking of 45 screened flavonoids, Table S9: Predicted pIC_50_ of screened substances against AChE and BACE-1, Table S10: Structures of 47 screened substances, Figure S1: ^1^H-NMR spectrum of F9, Figure S2: ^13^C-NMR spectrum of F9, Figure S3: ^1^H-NMR spectrum of F24, Figure S4: ^13^C-NMR spectrum of F24.
