# Supplementary material for: Design of Curcumin and Flavonoid Derivatives with Acetylcholinesterase and Beta-Secretase Inhibitory Activities Using in Silico Approaches
Source: Molecules. 2020 Aug 10;25(16):3644. doi: 10.3390/molecules25163644 (PMC7464027; doi:10.3390/molecules25163644)
Supplement: Supplementary file 1 [file molecules-25-03644-s001.pdf]

SUPPORTING INFORMATION

Design of Curcumin and Flavonoid Derivatives with Acetylcholinesterase and Beta-Secretase Inhibitory Activities Using in Silico Approaches

Thai-Son Tran [1,2,†], Minh-Tri Le [1,3,*,†] Thanh-Dao Tran [1], The-Huan Tran [2] and Khac-Minh Thai [1,*]

[1] Department of Medicinal Chemistry, Faculty of Pharmacy, University of Medicine and Pharmacy at Ho Chi Minh City, Ho Chi Minh City 700000, Vietnam; ttson@huemed-univ.edu.vn or tranthaison.hcmp@gmail.com (T-S.T.); tranthanhdao@uphcm.edu.vn (T-D.T.)

[2] Department of Pharmaceutical Chemistry, Faculty of Pharmacy, College of Medicine and Pharmacy, Hue University, Hue City 530000, Vietnam; tthuan@hueuni.edu.vn

[3] School of Medicine, Vietnam National University Ho Chi Minh City, 700000 Ho Chi Minh City, Vietnam

* Correspondence: leminhtri1099@gmail.com or leminhtri@ump.edu.vn (M-T.L.) and thaikhacminh@gmail.com or thaikhacminh@uphcm.edu.vn (K-M.T.); Tel.: +84-28-3855-2225 or +84-909-680-385 (K-M.T.); +84-903-718-190 (M-T.L.); Fax: +84-28-3822-5435 (K-M.T.)

† These authors contributed equally to this work.

Table S1. Pharmacophore model validation by goodness-of-hit score (GH) score method (AChE)

Table S2. Pharmacophore model validation by goodness-of-hit score (GH) score method (BACE-1)

Table S3. Dataset of 72 compounds used in the building of 2D-QSAR model for AChE inhibitors

Table S4. Dataset of 215 compounds used in the building of 2D-QSAR model for BACE-1 inhibitors

Table S5. List of 2D molecular descriptors computed using MOE 2008.10 software

Table S6. Re-docking results (RMSD values in Å)

Table S7. Results of Molecular Docking of Curcumins

Table S8. Results of Molecular Docking of 45 Screened Flavonoids

Table S9. Predicted pIC_{50} of screened substances against AChE and BACE-1

Table S10. Structures of 47 screened substances

Table S1. Pharmacophore model validation by goodness-of-hit score (GH) score method (AChE)

No.	Parameter	Pharmacophore Model												
		A1	A2	A3	A4	A5	A6	A7	A8	A9	A10	A11	A12	A13
1	Total molecules in database *(D)*	27024	27024	27024	27024	27024	27024	27024	27024	27024	27024	27024	27024	27024
2	Total number of actives in database *(A)*	655	655	655	655	655	655	655	655	655	655	655	655	655
3	Total hits *(Ht)*	914	1158	1115	1246	1121	1005	1269	882	1223	920	1367	945	944
4	Active Hits *(Ha)*	524	523	303	256	310	302	302	275	489	287	471	253	254
5	%Yield of actives *[(Ha/Ht)x100]*	57.33	45.16	27.17	20.55	27.65	30.05	23.80	31.18	39.98	31.20	34.46	26.77	26.91
6	%Ratio of actives *[(Ha/A) x 100]*	80.00	79.85	46.26	39.08	47.33	46.11	46.11	41.98	74.66	43.82	71.91	38.63	38.78
7	Enrichment factor *(E), [(Ha x D)/ (Ht x A)]*	23.65	18.63	11.21	8.48	11.41	12.40	9.82	12.86	16.50	12.87	14.22	11.05	11.10
8	False nagatives *[A-Ha]*	131	132	352	399	345	353	353	380	166	368	184	402	401
9	False positives *[Ht-Ha]*	390	635	812	990	811	703	967	607	734	633	896	692	690
10	Goodness of hit score *(GH)**	0.62	0.53	0.31	0.24	0.32	0.33	0.28	0.33	0.47	0.34	0.42	0.29	0.29

[(Ha/4HtA)(3A + Ht) x (1 – (Ht – Ha)/(D – A))]; GH score of 0.6-0.8 indicates a very good model

Table S2. Pharmacophore model validation by goodness-of-hit score (GH) score method (BACE-1)

No.	Parameters	Pharmacophore models										
		B1	B2	B3	B4	B5	B6	B7	B8	B9	B10	B11
1	Total molecules in database *(D)*	18653	18653	18653	18653	18653	18653	18653	18653	18653	18653	18653
2	Total number of actives in database *(A)*	436	436	436	436	436	436	436	436	436	436	436
3	Total hits *(Ht)*	438	964	876	895	833	801	644	1021	685	812	977
4	Active Hits *(Ha)*	305	221	284	237	190	304	241	277	229	278	280
5	%Yield of actives *[(Ha/Ht)x100]*	69.63	22.93	32.42	26.48	22.81	37.95	37.42	27.13	33.43	34.24	28.66
6	%Ratio of actives *[(Ha/A) x 100]*	69.95	50.69	65.14	54.36	43.58	69.72	55.28	63.53	52.52	63.76	64.22
7	Enrichment factor *(E), [(Ha x D)/ (Ht x A)]*	29.79	9.81	13.87	11.33	9.76	16.24	16.01	11.61	14.30	14.65	12.26
8	False nagatives *[A-Ha]*	131	215	152	199	246	132	195	159	207	158	156
9	False positives *[Ht-Ha]*	133	743	592	658	643	497	403	744	456	534	697
10	Goodness of hit score *(GH)**	0.69	0.29	0.39	0.32	0.27	0.45	0.41	0.35	0.37	0.40	0.36

[(Ha/4HtA)(3A + Ht) x (1 – (Ht – Ha)/(D – A))]; GH score of 0.6-0.8 indicates a very good model

Table S3. Dataset of 72 compounds used in the building of 2D-QSAR model for AChE inhibitors

No.	Name	pIC_{50}	predicted pIC_{50}
1	CHEMBL3133246	5.45	5.21
2	CHEMBL2296124	4.33	4.26
3	CHEMBL237223	4.59	4.72
4	CHEMBL2334727	4.72	4.60
5	CHEMBL3617370	4.51	4.42
6	CHEMBL3617380	4.49	4.46
7	CHEMBL2334728	4.24	4.28
8	CHEMBL416526	4.94	4.73
9	CHEMBL2393074	4.75	4.90
10	CHEMBL2385772	4.48	4.73
11	CHEMBL253386	5.49	5.22
12	CHEMBL3617382	4.63	4.52
13	CHEMBL3125443	4.90	4.67
14	CHEMBL3617375	4.28	4.49
15	CHEMBL3127216	4.84	4.70
16	CHEMBL3617367	4.38	4.27
17	CHEMBL457684	4.78	4.48
18	CHEMBL340807	4.80	4.66
19	CHEMBL2334726	4.49	4.55
20	CHEMBL659	5.62	5.42
21	CHEMBL2334730	4.64	4.78
22	CHEMBL3133247	4.81	5.07
23	CHEMBL517342	4.19	4.38
24	CHEMBL2296125	4.46	4.37
25	CHEMBL3617393	4.47	4.24
26	CHEMBL3617388	4.45	4.47
27	CHEMBL2385773	4.83	4.61
28	CHEMBL2385777	4.34	4.58
29	CHEMBL3127219	4.92	4.75
30	CHEMBL3127043	4.98	4.97
31	CHEMBL1269160	4.57	4.42
32	CHEMBL2334725	4.70	4.76
33	CHEMBL1270150	4.73	4.81
34	CHEMBL500712	4.40	4.21
35	CHEMBL2334736	4.72	4.83
36	CHEMBL2018160	4.63	4.48
37	CHEMBL2334737	4.71	4.61
38	CHEMBL269538	4.19	4.49
39	CHEMBL3617397	4.52	4.32

40	CHEMBL2334745	4.49	4.70
41	CHEMBL2296112	4.25	4.44
42	CHEMBL3617381	4.49	4.53
43	CHEMBL3133250	4.29	4.63
44	CHEMBL12014	4.23	4.50
45	CHEMBL3617372	4.26	4.44
46	CHEMBL3133243	4.97	5.10
47	CHEMBL510090	4.72	4.82
48	CHEMBL2385771	4.30	4.56
49	CHEMBL2296117	4.18	4.13
50	CHEMBL3127217	4.99	4.86
51	CHEMBL2334738	4.69	4.66
52	CHEMBL2296113	4.21	4.40
53	CHEMBL2381433	4.58	4.53
54	CHEMBL3132866	4.98	5.01
55	CHEMBL2385768	4.75	4.66
56	CHEMBL2385780	4.88	4.68
57	CHEMBL2334739	4.52	4.35
58	CHEMBL3133245	5.36	5.17
59	CHEMBL1270255	5.01	5.24
60	CHEMBL3133248	4.66	4.90
61	CHEMBL3617362	4.22	4.42
62	CHEMBL2381434	4.67	4.49
63	CHEMBL1535235	4.61	4.53
64	CHEMBL129177	4.47	4.70
65	CHEMBL2385784	4.53	4.76
66	CHEMBL2385779	4.42	4.23
67	CHEMBL3617396	4.39	4.26
68	CHEMBL1085869	5.67	5.51
69	CHEMBL2334747	4.51	4.56
70	CHEMBL2385766	4.35	4.57
71	CHEMBL463856	4.33	4.26
72	CHEMBL2334732	4.86	4.81

Table S4. Dataset of 215 compounds used in the building of 2D-QSAR model for BACE-1 inhibitors

No.	Name	pIC_{50}	Predicted pIC_{50}
1	BMC-2009-3671-17	6.92	7.02
2	BMC-2004-247-8	6.46	6.57
3	BMC-2007-1023-8	8.30	7.97
4	JMC-2012-27-30	5.49	5.65
5	BMC-2004-248-21	7.17	6.96
6	BMC-2009-3675-11	7.96	8.19
7	BMC-2009-3671-20	6.82	7.33
8	BMC-2009-3677-26	7.49	7.93
9	BMC-2008-1019-14	6.26	6.86
10	BMC-2014-2038-39	7.74	7.51
11	BMC-2013-4677-30	6.83	6.54
12	BMC-2004-248-19	7.37	6.74
13	BMC-2013-4677-37	5.88	6.33
14	BMC-2013-4241-10	6.46	6.45
15	BMC-2014-2036-24	7.49	8.02
16	BMC-2013-4675-7	7.35	6.70
17	BMC-2009-3667-16	7.72	7.63
18	BMC-2014-2039-43	7.28	7.39
19	EODD-2013-723-102	6.62	6.99
20	JMC-2012-26-15	5.00	5.15
21	JMC-2012-27-37	5.70	5.84
22	EODD-2013-718-42c	8.00	8.56
23	JMC-2012-9013-24	7.11	7.90
24	BMC-2014-2041-65	6.24	6.08
25	BMC-2008-1021-30	6.48	7.12
26	BMC-2009-3672-21	8.10	7.85
27	JMC-2012-9013-26	7.58	7.52
28	EODD-2013-720-53	6.82	7.20
29	BMC-2008-1021-31	6.64	6.66
30	BMC-2008-1021-28	7.08	6.58
31	JMC-2012-27-41	6.20	6.58
32	BMC-2009-3675-7	8.40	8.17
33	BMC-2009-3673-39	7.70	7.35
34	BMC-2014-2042-71	6.62	6.31
35	EODD-2013-713-11	7.55	7.25
36	BMC-2009-3672-27	7.04	7.46
37	BMC-2014-2042-90	8.07	7.85
38	EODD-2013-719-48	8.48	8.21
39	JMC-2012-27-47	6.49	6.45

40	BMC_2014-2042-81	7.60	8.12
41	BMC-2009-3665-4	8.04	7.43
42	BMC-2009-3673-36	7.64	7.69
43	BMC-2013-4676-17	6.93	7.33
44	BMC-2014-2038-41	7.70	7.60
45	BMC-2013-4677-38	6.07	6.44
46	BMC-2009-3667-20	7.47	7.71
47	EODD-2013-720-58	7.70	7.39
48	JMC-2012-26-24b	6.20	5.74
49	BMC-2014-2036-23	7.70	7.56
50	BMC-2013-4677-53	7.05	7.14
51	BMC-2007-1023-2	6.60	7.23
52	BMC-2009-3667-17	7.64	7.27
53	BMC-2008-1020-23	6.22	6.37
54	BMC-2014-2041-66	6.15	6.14
55	BMC-2013-4241-4	5.99	6.39
56	BMC-2009-3673-34	7.85	7.19
57	BMC-2014-2036-17	7.32	7.35
58	BMC-2008-1020-18	6.12	5.95
59	BMC-2014-2038-37	4.66	5.00
60	BMC-2009-3672-24	8.30	7.96
61	BMC-2007-1024-18	7.40	7.93
62	BMC-2008-1020-24	6.80	6.66
63	JMC-2012-28-64	6.10	6.08
64	BMC-2014-2035-13	8.00	7.51
65	BMC-2014-2040-53	8.15	8.02
66	BMC-2009-3667-18a	7.58	7.41
67	BMC-2009-3666-10	7.64	8.03
68	JMC-2012-9010-17	8.10	7.96
69	BMC-2014-2036-20	7.11	7.79
70	EODD-2013-721-64	6.33	6.65
71	BMC-2013-4677-35	6.38	6.60
72	BMC-2009-3671-12	7.04	7.62
73	EODD-2013-718-44	8.70	8.08
74	EODD-2013-721-65	7.20	6.65
75	BMC-2013-4242-23	7.34	7.00
76	BMC-2013-4241-9	7.92	7.22
77	BMC-2014-2040-62	7.69	7.61
78	BMC-2007-1023-5	7.30	7.87
79	BMC-2004-247-9	6.26	6.30
80	EODD-2013-718-42b	8.04	8.33

81	BMC-2009-3676-18	8.10	7.76
82	BMC-2007-1023-6	8.22	8.01
83	BMC-2009-3668-25	8.22	7.78
84	EODD-2013-718-45	7.68	7.34
85	BMC-2004-246-4	6.72	6.46
86	BMC-2013-4241-14	7.51	7.15
87	BMC-2009-3667-19	7.55	7.59
88	BMC-2007-1025-22	8.22	7.68
89	EODD-2013-716-31	8.70	8.27
90	BMC-2014-2042-85	6.53	7.06
91	BMC-2007-1023-11	8.30	8.05
92	BMC-2009-3671-16	7.58	8.09
93	BMC-2014-2039-42	7.08	7.22
94	BMC-2009-3666-11	7.74	8.32
95	BMC-2014-2038-40	7.70	7.27
96	BMC-2004-248-13	6.68	7.04
97	BMC-2014-2039-47	7.57	7.82
98	BMC-2009-3672-25	7.22	7.43
99	BMC-2009-3677-23	7.30	7.25
100	BMC-2009-3666-7	7.20	7.33
101	JMC-2012-28-83	5.10	5.52
102	BMC-2014-2035-14	7.70	8.20
103	BMC-2007-1023-10	8.30	8.19
104	BMC-2014-2035-15	8.00	8.16
105	BMC-2014-2036-22	7.04	6.54
106	EODD-2013-718-42a	7.89	8.20
107	BMC-2014-2042-72	7.58	7.34
108	BMC-2013-4242-24	6.43	6.98
109	BMC-2009-3672-26	8.04	7.58
110	BMC-2009-3676-21	6.60	6.71
111	BMC-2009-3677-24	7.40	7.21
112	BMC-2013-4242-20	6.92	6.62
113	BMC-2009-3675-9	8.00	7.47
114	BMC-2013-4241-5	6.26	6.22
115	JMC-2012-26-19b	5.20	5.44
116	BMC-2014-2042-87	7.31	7.24
117	BMC-2014-2042-84	6.49	7.03
118	BMC-2013-4676-19	7.38	7.76
119	BMC-2013-4241-3	5.05	5.61
120	BMC-2009-3668-27	7.40	7.26
121	BMC-2009-3677-25	7.70	7.26

122	BMC-2009-3675-10	7.66	7.50
123	BMC-2007-1025-21	8.40	7.77
124	BMC-2007-1024-17	8.30	8.17
125	BMC-2004-246-5	6.89	6.25
126	BMC-2009-3676-19	7.06	7.16
127	BMC-2013-4675-4	6.24	6.39
128	BMC-2014-2041-64	6.92	6.23
129	BMC-2004-246-6	5.95	6.41
130	BMC-2013-4242-22	7.64	7.14
131	BMC-2007-1024-15	7.92	7.44
132	BMC-2013-4241-12	6.86	6.99
133	BMC-2009-3673-35	8.30	7.86
134	BMC-2008-1019-13	6.52	6.58
135	BMC-2013-4241-16	6.12	6.63
136	BMC-2014-2042-73	7.52	7.45
137	JMC-2012-27-34	5.70	5.91
138	BMC-2013-4677-36	6.65	6.43
139	BMC-2004-248-20	6.75	6.64
140	BMC-2009-3672-23	7.64	7.81
141	JMC-2012-9010-18	7.64	7.67
142	BMC-2009-3675-13	7.64	7.55
143	JMC-2012-28-65	6.30	6.03
144	BMC-2013-4241-15	7.35	7.51
145	BMC-2004-246-7	5.76	6.23
146	BMC-2013-4677-48	8.04	7.86
147	BMC-2013-4241-13	7.41	7.34
148	BMC-2013-4675-6	6.42	6.42
149	BMC-2009-3671-9	8.70	8.29
150	BMC-2009-3671-11	7.72	8.26
151	BMC-2008-1019-12	7.48	6.96
152	BMC-2008-1021-27	7.70	7.30
153	BMC-2014-2039-46	7.27	7.16
154	BMC-2014-2035-9	7.10	6.33
155	JMC-2012-9010-15	7.74	7.98
156	BMC-2007-1024-13	7.47	7.41
157	BMC-2014-2042-79	7.40	7.59
158	EODD-2013-716-32	8.40	8.23
159	BMC-2008-1021-29	7.00	7.42
160	JMC-2012-9013-25	7.48	7.95
161	BMC-2004-246-3	7.09	7.26
162	BMC-2014-2042-80	7.47	7.90

163	BMC-2004-247-10	6.46	6.20
164	JMC-2012-9013-23	7.54	8.48
165	BMC-2008-1020-26	7.85	7.24
166	BMC-2009-3667-18b	7.40	7.51
167	EODD-2013-713-12a	8.41	8.84
168	BMC-2009-3677-27	7.10	7.58
169	BMC-2004-248-14	6.68	6.49
170	BMC-2007-1024-12	6.92	6.75
171	EODD-2013-718-41	7.30	7.95
172	BMC-2009-3675-14	7.32	7.60
173	BMC-2008-1019-8	5.61	6.20
174	BMC-2008-1020-22	5.77	6.13
175	BMC-2008-1019-6	6.74	6.26
176	BMC-2009-3671-15	8.15	8.22
177	BMC-2013-4242-19	7.21	6.91
178	BMC-2014-2042-82	7.55	7.36
179	EODD-2013-721-61	6.96	7.39
180	BMC-2008-1020-16	7.40	7.28
181	BMC-2014-2042-76	8.70	8.94
182	BMC-2009-3675-5	8.30	7.93
183	BMC-2009-3675-16	7.70	7.51
184	BMC-2007-1023-9	7.96	8.00
185	BMC-2014-2042-78	7.70	7.43
186	BMC-2008-1019-7	5.95	6.35
187	BMC-2013-4675-3	6.27	6.20
188	JMC-2012-26-8b	4.60	4.57
189	EODD-2013-713-10	7.06	7.07
190	JMC-2012-26-19a	5.00	5.44
191	BMC-2014-2035-12	5.47	5.08
192	BMC-2013-4676-18	6.90	7.35
193	JMC-2012-9013-19	8.27	8.63
194	BMC-2004-248-17	7.22	6.58
195	BMC-2014-2040-54	8.22	8.06
196	BMC-2009-3671-13	7.82	7.98
197	JMC-2012-26-24a	6.00	6.04
198	BMC-2008-1020-19	7.77	7.61
199	EODD-2013-719-50	7.77	8.60
200	JMC-2012-27-54a	5.40	5.90
201	EODD-2013-717-39a	9.40	9.53
202	BMC-2007-1024-14	7.74	8.09
203	BMC-2013-4677-31	6.73	6.68

204	BMC-2009-3672-22	8.70	8.02
205	BMC-2009-3667-23	6.38	6.32
206	BMC-2013-4675-5	7.03	6.31
207	BMC-2013-4677-32	7.22	7.24
208	BMC-2014-2042-88	7.44	7.03
209	BMC-2007-1023-7	8.52	7.97
210	JMC-2012-26-8a	4.79	4.94
211	JMC-2012-9013-22	7.69	8.31
212	BMC-2013-4241-18	7.02	6.58
213	BMC-2014-2042-75	8.00	8.54
214	JMC-2012-9010-16	7.45	8.05
215	BMC-2007-1024-16	8.10	7.53

Table S5. List of 2D molecular descriptors computed using MOE 2008.10 software

Code of descriptor	Description
**Physical Properties Descriptors**	
apol	Sum of the atomic polarizabilities (including implicit hydrogens) with polarizabilities taken from [CRC 1994].
bpol	Sum of the absolute value of the difference between atomic polarizabilities of all bonded atoms in the molecule (including implicit hydrogens) with polarizabilities taken from [CRC 1994].
density	Molecular mass density: Weight divided by vdw_vol (amu/Å^3).
FCharge	Total charge of the molecule (sum of formal charges).
mr	Molecular refractivity (including implicit hydrogens). This property is calculated from an 11 descriptor linear model [MREF 1998] with r^2 = 0.997, RMSE = 0.168 on 1,947 small molecules.
SMR	Molecular refractivity (including implicit hydrogens). This property is an atomic contribution model [Crippen 1999] that assumes the correct protonation state (washed structures). The model was trained on ~7000 structures and results may vary from the mr descriptor.
Weight	Molecular weight (including implicit hydrogens) in atomic mass units with atomic weights taken from [CRC 1994].
logP(o/w)	Log of the octanol/water partition coefficient (including implicit hydrogens). This property is calculated from a linear atom type model [LOGP 1998] with r^2 = 0.931, RMSE = 0.393 on 1,827 molecules.
logS	Log of the aqueous solubility (mol/L). This property is calculated from an atom contribution linear atom type model [Hou 2004] with r^2 = 0.90, ~1,200 molecules.
reactive	Indicator of the presence of reactive groups. A non-zero value indicates that the molecule contains a reactive group. The table of reactive groups is based on the Oprea set [Oprea 2000] and includes metals, phospho-, N/O/S-N/O/S single bonds, thiols, acyl halides, Michael Acceptors, azides, esters, etc.
SlogP	Log of the octanol/water partition coefficient (including implicit hydrogens). This property is an atomic contribution model [Crippen 1999] that calculates logP from the given structure; i.e., the correct protonation state (washed structures). Results may vary from the logP(o/w) descriptor. The training set for SlogP was ~7000 structures.
TPSA	Polar surface area (Å^2) calculated using group contributions to approximate the polar surface area from connection table information only. The parameterization is that of Ertl *et al.* [Ertl 2000].
vdw_vol	*van der Waals* volume (Å^3) calculated using a connection table approximation.
vdw_area	Area of *van der Waals* surface (Å^2) calculated using a connection table approximation.
**Subdivided Surface Areas Descriptors**<br>The Subdivided Surface Areas are descriptors based on an approximate accessible *van der Waals* surface area (in Å^2) calculation for each atom, v_i along with some other atomic property, p_i. The v_i are calculated using a connection table approximation. Each descriptor in a series is defined to be the sum of the v_i over all atoms i such that p_i is in a specified range (a,b).<br>In the descriptions to follow, L_i denotes the contribution to logP(o/w) for atom i as calculated in the SlogP descriptor [Crippen 1999]. R_i denotes the contribution to Molar Refractivity for atom i as calculated in the SMR descriptor [Crippen 1999]. The ranges were determined by percentile subdivision over a large collection of compounds.	
SlogP_VSA0	Sum of v_i such that L_i <= -0.4.
SlogP_VSA1	Sum of v_i such that L_i is in (-0.4,-0.2].
SlogP_VSA2	Sum of v_i such that L_i is in (-0.2,0].
SlogP_VSA3	Sum of v_i such that L_i is in (0,0.1].
SlogP_VSA4	Sum of v_i such that L_i is in (0.1,0.15].
SlogP_VSA5	Sum of v_i such that L_i is in (0.15,0.20].
SlogP_VSA6	Sum of v_i such that L_i is in (0.20,0.25].

SlogP_VSA7	Sum of v_i such that L_i is in (0.25,0.30].
SlogP_VSA8	Sum of v_i such that L_i is in (0.30,0.40].
SlogP_VSA9	Sum of v_i such that $L_i > 0.40$.
SMR_VSA0	Sum of v_i such that R_i is in [0,0.11].
SMR_VSA1	Sum of v_i such that R_i is in (0.11,0.26].
SMR_VSA2	Sum of v_i such that R_i is in (0.26,0.35].
SMR_VSA3	Sum of v_i such that R_i is in (0.35,0.39].
SMR_VSA4	Sum of v_i such that R_i is in (0.39,0.44].
SMR_VSA5	Sum of v_i such that R_i is in (0.44,0.485].
SMR_VSA6	Sum of v_i such that R_i is in (0.485,0.56].
SMR_VSA7	Sum of v_i such that $R_i > 0.56$.
**Atom Counts and Bond Counts Descriptors**<br>Z denotes the *atomic number* of an atom; lone pair pseudo-atoms (LP) are given an atomic number of 0. *Heavy atoms* are atoms that have an atomic number strictly greater than 1 (not H nor LP). A *trivial atom* is an LP pseudo-atom or a hydrogen with exactly one heavy neighbor.<br>The *hydrogen count*, h, of an atom is the number of hydrogens to which it is (or should be) attached. This count includes all hydrogen atoms that are necessary to fill valence.<br>The *heavy degree, d*, of an atom is the number of heavy atoms to which it is bonded. That is, d is the number of bonded neighbors of the atom in the hydrogen suppressed graph.	
a_aro	Number of aromatic atoms.
a_count	Number of atoms (including implicit hydrogens). This is calculated as the sum of $(1 + h_i)$ over all non-trivial atoms i.
a_heavy	Number of heavy atoms $\#\{Z_i \mid Z_i > 1\}$.
a_ICM	Atom information content (mean). This is the entropy of the element distribution in the molecule (including implicit hydrogens but not lone pair pseudo-atoms). Let n_i be the number of occurrences of atomic number i in the molecule.<br>Let $p_i = n_i / n$ where n is the sum of the n_i. The value of a_ICM is the negative of the sum over all i of $p_i \log p_i$.
a_IC	Atom information content (total). This is calculated to be a_ICM times n.
a_nH	Number of hydrogen atoms (including implicit hydrogens). This is calculated as the sum of h_i over all non-trivial atoms i plus the number of non-trivial hydrogen atoms.
a_nB	Number of boron atoms: $\#\{Z_i \mid Z_i = 5\}$.
a_nC	Number of carbon atoms: $\#\{Z_i \mid Z_i = 6\}$.
a_nN	Number of nitrogen atoms: $\#\{Z_i \mid Z_i = 7\}$.
a_nO	Number of oxygen atoms: $\#\{Z_i \mid Z_i = 8\}$.
a_nF	Number of fluorine atoms: $\#\{Z_i \mid Z_i = 9\}$.
a_nP	Number of phosphorus atoms: $\#\{Z_i \mid Z_i = 15\}$.
a_nS	Number of sulfur atoms: $\#\{Z_i \mid Z_i = 16\}$.
a_nCl	Number of chlorine atoms: $\#\{Z_i \mid Z_i = 17\}$.
a_nBr	Number of bromine atoms: $\#\{Z_i \mid Z_i = 35\}$.
a_nI	Number of iodine atoms: $\#\{Z_i \mid Z_i = 53\}$.
b_1rotN	Number of rotatable single bonds. Conjugated single bonds are not included (e.g., ester and peptide bonds).
b_1rotR	Fraction of rotatable single bonds: b_1rotN divided by b_heavy.
b_ar	Number of aromatic bonds.
b_count	Number of bonds (including implicit hydrogens). This is calculated as the sum of $(d_i/2 + h_i)$ over all non-trivial atoms i.
b_double	Number of double bonds. Aromatic bonds are not considered to be double bonds.
b_heavy	Number of bonds between heavy atoms.
b_rotN	Number of rotatable bonds. A bond is rotatable if it has order 1, is not in a ring, and has at least two heavy neighbors.
b_rotR	Fraction of rotatable bonds: b_rotN divided by b_heavy.
b_single	Number of single bonds (including implicit hydrogens). Aromatic bonds are not considered to be single bonds.

b_triple	Number of triple bonds. Aromatic bonds are not considered to be triple bonds.
chiral	The number of chiral centers.
chiral_u	The number of unconstrained chiral centers.
lip_acc	The number of O and N atoms.
lip_don	The number of OH and NH atoms.
lip_druglike	One if and only if lip_violation < 2 otherwise zero.
lip_violation	The number of violations of Lipinski's Rule of Five [Lipinski 1997].
nmol	The number of molecules (connected components).
opr_brigid	The number of rigid bonds from [Oprea 2000].
opr_leadlike	One if and only if opr_violation < 2 otherwise zero.
opr_nring	The number of ring bonds from [Oprea 2000].
opr_nrot	The number of rotatable bonds from [Oprea 2000].
opr_violation	The number of violations of Oprea's lead-like test [Oprea 2000].
rings	The number of rings.
VAdjMa	Vertex adjacency information (magnitude): $1 + \log_2 m$ where m is the number of heavy-heavy bonds. If m is zero, then zero is returned.
VAdjEq	Vertex adjacency information (equality): $-(1-f)\log_2(1-f) - f \log_2 f$ where $f = (n^2 - m) / n^2$, n is the number of heavy atoms and m is the number of heavy-heavy bonds. If f is not in the open interval (0,1), then 0 is returned.

Kier&Hall Connectivity and Kappa Shape Indices Descriptors
For a heavy atom i let $v_i = (p_i - h_i) / (Z_i - p_i - 1)$ where p_i is the number of s and p valence electrons of atom i. The Kier and Hall chi connectivity indices are calculated from the heavy atom degree d_i (number of heavy neighbors) and v_i. The Kier and Hall kappa molecular shape indices [Hall 1991] compare the molecular graph with minimal and maximal molecular graphs, and are intended to capture different aspects of molecular shape. In the following description, n denotes the number of atoms in the hydrogen suppressed graph, m is the number of bonds in the hydrogen suppressed graph and a is the sum of $(r_i/r_c - 1)$ where r_i is the covalent radius of atom i, and r_c is the covalent radius of a carbon atom. Also, let p_2 denote the number of paths of length 2 and p_3 the number of paths of length 3.

chi0	Atomic connectivity index (order 0) from [Hall 1991] and [Hall 1977]. This is calculated as the sum of $1/\text{sqrt}(d_i)$ over all heavy atoms i with $d_i > 0$.
chi0_C	Carbon connectivity index (order 0). This is calculated as the sum of $1/\text{sqrt}(d_i)$ over all carbon atoms i with $d_i > 0$.
chi1	Atomic connectivity index (order 1) from [Hall 1991] and [Hall 1977]. This is calculated as the sum of $1/\text{sqrt}(d_i d_j)$ over all bonds between heavy atoms i and j where $i < j$.
chi1_C	Carbon connectivity index (order 1). This is calculated as the sum of $1/\text{sqrt}(d_i d_j)$ over all bonds between carbon atoms i and j where $i < j$.
chi0v	Atomic valence connectivity index (order 0) from [Hall 1991] and [Hall 1977]. This is calculated as the sum of $1/\text{sqrt}(v_i)$ over all heavy atoms i with $v_i > 0$.
chi0v_C	Carbon valence connectivity index (order 0). This is calculated as the sum of $1/\text{sqrt}(v_i)$ over all carbon atoms i with $v_i > 0$.
chi1v	Atomic valence connectivity index (order 1) from [Hall 1991] and [Hall 1977]. This is calculated as the sum of $1/\text{sqrt}(v_i v_j)$ over all bonds between heavy atoms i and j where $i < j$.
chi1v_C	Carbon valence connectivity index (order 1). This is calculated as the sum of $1/\text{sqrt}(v_i v_j)$ over all bonds between carbon atoms i and j where $i < j$.
Kier1	First kappa shape index: $(n-1)^2 / m^2$ [Hall 1991].
Kier2	Second kappa shape index: $(n-1)^2 / m^2$ [Hall 1991].
Kier3	Third kappa shape index: $(n-1)(n-3)^2 / p_3^2$ for odd n, and $(n-3)(n-2)^2 / p_3^2$ for even n [Hall 1991].
KierA1	First alpha modified shape index: $s(s-1)^2 / m^2$ where $s = n + a$ [Hall 1991].
KierA2	Second alpha modified shape index: $s(s-1)^2 / m^2$ where $s = n + a$ [Hall 1991].
KierA3	Third alpha modified shape index: $(n-1)(n-3)^2 / p_3^2$ for odd n, and $(n-3)(n-2)^2 / p_3^2$ for even n where $s = n + a$ [Hall 1991].

KierFlex	Kier molecular flexibility index: (KierA1) (KierA2) / *n* [Hall 1991].
zagreb	Zagreb index: the sum of d_i^2 over all heavy atoms *i*.
**Adjacency and Distance Matrix Descriptors**<br>The *adjacency matrix*, *M*, of a chemical structure is defined by the elements [M_{ij}] where M_{ij} is 1 if atoms *i* and *j* are bonded and zero otherwise. The *distance matrix*, *D*, of a chemical structure is defined by the elements [D_{ij}] where D_{ij} is the length of the shortest path from atoms *i* to *j*; zero is used if atoms *i* and *j* are not part of the same connected component.	
balabanJ	Balaban's connectivity topological index [Balaban 1982].
BCUT_PEOE_0<br>BCUT_PEOE_1<br>BCUT_PEOE_2<br>BCUT_PEOE_3	The BCUT descriptors [Pearlman 1998] are calculated from the eigenvalues of a modified adjacency matrix. Each *ij* entry of the adjacency matrix takes the value 1/sqrt(b_{ij}) where b_{ij} is the formal bond order between bonded atoms *i* and *j*. The diagonal takes the value of the PEOE partial charges. The resulting eigenvalues are sorted and the smallest, 1/3-ile, 2/3-ile and largest eigenvalues are reported.
BCUT_SLOGP_0<br>BCUT_SLOGP_1<br>BCUT_SLOGP_2<br>BCUT_SLOGP_3	The BCUT descriptors using atomic contribution to logP (using the Wildman and Crippen SlogP method) instead of partial charge.
BCUT_SMR_0<br>BCUT_SMR_1<br>BCUT_SMR_2<br>BCUT_SMR_3	The BCUT descriptors using atomic contribution to molar refractivity (using the Wildman and Crippen SMR method) instead of partial charge.
diameter	Largest value in the distance matrix [Petitjean 1992].
petitjean	Value of (diameter - radius) / diameter.
GCUT_PEOE_0<br>GCUT_PEOE_1<br>GCUT_PEOE_2<br>GCUT_PEOE_3	The GCUT descriptors are calculated from the eigenvalues of a modified graph distance adjacency matrix. Each *ij* entry of the adjacency matrix takes the value 1/sqr(d_{ij}) where d_{ij} is the (modified) graph distance between atoms *i* and *j*. The diagonal takes the value of the PEOE partial charges. The resulting eigenvalues are sorted and the smallest, 1/3-ile, 2/3-ile and largest eigenvalues are reported.
GCUT_SLOGP_0<br>GCUT_SLOGP_1<br>GCUT_SLOGP_2<br>GCUT_SLOGP_3	The GCUT descriptors using atomic contribution to logP (using the Wildman and Crippen SlogP method) instead of partial charge.
GCUT_SMR_0<br>GCUT_SMR_1<br>GCUT_SMR_2<br>GCUT_SMR_3	The GCUT descriptors using atomic contribution to molar refractivity (using the Wildman and Crippen SMR method) instead of partial charge.
petitjeanSC	Petitjean graph Shape Coefficient as defined in [Petitjean 1992]: (diameter - radius) / radius.
radius	If r_i is the largest matrix entry in row *i* of the distance matrix *D*, then the radius is defined as the smallest of the r_i [Petitjean 1992].
VDistEq	If *m* is the sum of the distance matrix entries then VdistEq is defined to be the sum of $\log_2 m - p_i \log_2 p_i / m$ where p_i is the number of distance matrix entries equal to *i*.
VDistMa	If *m* is the sum of the distance matrix entries then VDistMa is defined to be the sum of $\log_2 m - D_{ij} \log_2 D_{ij} / m$ over all *i* and *j*.
wienerPath	Wiener path number: half the sum of all the distance matrix entries as defined in [Balaban 1979] and [Wiener 1947].
wienerPol	Wiener polarity number: half the sum of all the distance matrix entries with a value of 3 as defined in [Balaban 1979].
**Pharmacophore Feature Descriptors**	
a_acc	Number of hydrogen bond acceptor atoms (not counting acidic atoms but counting atoms that are both hydrogen bond donors and acceptors such as -OH).
a_acid	Number of acidic atoms.
a_base	Number of basic atoms.
a_don	Number of hydrogen bond donor atoms (not counting basic atoms but counting atoms that are both hydrogen bond donors and acceptors such as -OH).

a_hyd	Number of hydrophobic atoms.
vsa_acc	Approximation to the sum of VDW surface areas (Å^2) of pure hydrogen bond acceptors (not counting acidic atoms and atoms that are both hydrogen bond donors and acceptors such as -OH).
vsa_acid	Approximation to the sum of VDW surface areas of acidic atoms (Å^2).
vsa_base	Approximation to the sum of VDW surface areas of basic atoms (Å^2).
vsa_don	Approximation to the sum of VDW surface areas of pure hydrogen bond donors (not counting basic atoms and atoms that are both hydrogen bond donors and acceptors such as -OH) (Å^2).
vsa_hyd	Approximation to the sum of VDW surface areas of hydrophobic atoms (Å^2).
vsa_other	Approximation to the sum of VDW surface areas (Å^2) of atoms typed as "other".
vsa_pol	Approximation to the sum of VDW surface areas (Å^2) of polar atoms (atoms that are both hydrogen bond donors and acceptors), such as -OH.
**Partial Charge Descriptors**<br>Descriptors that depend on the partial charge of each atom of a chemical structure require calculation of those partial charges. Let q_i denote the partial charge of atom *i* as defined above. Let v_i be the *van der Waals* surface area (Å^2) of atom *i* (as calculated by a connection table approximation). The following descriptors are calculated:	
Q_PC+<br>PEOE_PC+	Total positive partial charge: the sum of the positive q_i. Q_PC+ is identical to PC+ which has been retained for compatibility.
Q_PC-<br>PEOE_PC-	Total negative partial charge: the sum of the negative q_i. Q_PC- is identical to PC- which has been retained for compatibility.
Q_RPC+<br>PEOE_RPC+	Relative positive partial charge: the largest positive q_i divided by the sum of the positive q_i. Q_RPC+ is identical to RPC+ which has been retained for compatibility.
Q_PRC-<br>PEOE_RPC-	Relative negative partial charge: the smallest negative q_i divided by the sum of the negative q_i. Q_RPC- is identical to RPC- which has been retained for compatibility.
Q_VSA_POS<br>PEOE_VSA_POS	Total positive *van der Waals* surface area. This is the sum of the v_i such that q_i is non-negative. The v_i are calculated using a connection table approximation.
Q_VSA_NEG<br>PEOE_VSA_NEG	Total negative *van der Waals* surface area. This is the sum of the v_i such that q_i is negative. The v_i are calculated using a connection table approximation.
Q_VSA_PPOS<br>PEOE_VSA_PPOS	Total positive polar *van der Waals* surface area. This is the sum of the v_i such that q_i is greater than 0.2. The v_i are calculated using a connection table approximation.
Q_VSA_PNEG<br>PEOE_VSA_PNEG	Total negative polar *van der Waals* surface area. This is the sum of the v_i such that q_i is less than -0.2. The v_i are calculated using a connection table approximation.
Q_VSA_HYD<br>PEOE_VSA_HYD	Total hydrophobic *van der Waals* surface area. This is the sum of the v_i such that $\|q_i\|$ is less than or equal to 0.2. The v_i are calculated using a connection table approximation.
Q_VSA_POL<br>PEOE_VSA_POL	Total polar *van der Waals* surface area. This is the sum of the v_i such that $\|q_i\|$ is greater than 0.2. The v_i are calculated using a connection table approximation.
Q_VSA_FPOS<br>PEOE_VSA_FPOS	Fractional positive *van der Waals* surface area. This is the sum of the v_i such that q_i is non-negative divided by the total surface area. The v_i are calculated using a connection table approximation.
Q_VSA_FNEG<br>PEOE_VSA_FNEG	Fractional negative *van der Waals* surface area. This is the sum of the v_i such that q_i is negative divided by the total surface area. The v_i are calculated using a connection table approximation.
Q_VSA_FPPOS<br>PEOE_VSA_FPPOS	Fractional positive polar *van der Waals* surface area. This is the sum of the v_i such that q_i is greater than 0.2 divided by the total surface area. The v_i are calculated using a connection table approximation.
Q_VSA_FPNEG<br>PEOE_VSA_FPNEG	Fractional negative polar *van der Waals* surface area. This is the sum of the v_i such that q_i is less than -0.2 divided by the total surface area. The v_i are calculated using a connection table approximation.
Q_VSA_FHYD<br>PEOE_VSA_FHYD	Fractional hydrophobic *van der Waals* surface area. This is the sum of the v_i such that $\|q_i\|$ is less than or equal to 0.2 divided by the total surface area. The v_i are calculated using a connection table approximation.
Q_VSA_FPOL	Fractional polar *van der Waals* surface area. This is the sum of the v_i such that $\|q_i\|$ is

PEOE_VSA_FPOL	greater than 0.2 divided by the total surface area. The v_i are calculated using a connection table approximation.
PEOE_VSA+6	Sum of v_i where q_i is greater than 0.3.
PEOE_VSA+5	Sum of v_i where q_i is in the range [0.25,0.30).
PEOE_VSA+4	Sum of v_i where q_i is in the range [0.20,0.25).
PEOE_VSA+3	Sum of v_i where q_i is in the range [0.15,0.20).
PEOE_VSA+2	Sum of v_i where q_i is in the range [0.10,0.15).
PEOE_VSA+1	Sum of v_i where q_i is in the range [0.05,0.10).
PEOE_VSA+0	Sum of v_i where q_i is in the range [0.00,0.05).
PEOE_VSA-0	Sum of v_i where q_i is in the range [-0.05,0.00).
PEOE_VSA-1	Sum of v_i where q_i is in the range [-0.10,-0.05).
PEOE_VSA-2	Sum of v_i where q_i is in the range [-0.15,-0.10).
PEOE_VSA-3	Sum of v_i where q_i is in the range [-0.20,-0.15).
PEOE_VSA-4	Sum of v_i where q_i is in the range [-0.25,-0.20).
PEOE_VSA-5	Sum of v_i where q_i is in the range [-0.30,-0.25).
PEOE_VSA-6	Sum of v_i where q_i is less than -0.30.

Table S6. Re-docking results (RMSD values in Å)

Ligand	AChE							
	1ACJ	1DX6	1EVE	1W6R	4EY6 (chain A)	4EY6 (chain B)	4EY7 (chain A)	4EY7 (chain B)
1	0.52	0.49	0.69	0.49	0.35	0.54	1.14	0.96
2	0.42	0.50	0.87	0.45	0.71	0.54	1.70	1.58
3	0.49	0.75	0.99	0.62	0.46	0.72	1.17	1.10
**Ligand**	**BACE-1**							
	3VEU	4B78	5HTZ	5HU0 (chain A)	5HU0 (chain B)	5HU1 (chain A)	5HU1 (chain B)	
1	1.12	0.83	2.09	1.19	1.48	0.75	0.97	
2	1.14	0.80	2.93	1.34	1.01	1.16	1.29	
3	1.47	0.92	2.67	1.62	1.01	1.15	1.41	

- Ligand 1: separated from the complex (native form, not prepared).

- Ligand 2: separated from the complex and re-prepared using mentioned appropriate procedure.

- Ligand 3: built and prepared from the beginning.

Table S7. Results of Molecular Docking of Curcumins

AChE

Comp.	Docking score (kJmol^{-1})							
	1ACJ	1DX6	1EVE	1W6R	4EY6 (chain A)	4EY6 (chain B)	4EY7 (chain A)	4EY7 (chain B)
C1	Not docked	-24.1328	-25.6212	-25.4662	Not docked	Not docked	-34.3784	-36.2289
C2	Not docked	-30.9742	-23.9748	-25.1888	-23.2125	-12.7174	-23.5571	-31.9032

BACE-1

Comp.	Docking score (kJmol^{-1})					
	3VEU	4B78	5HU0 (chain A)	5HU0 (chain B)	5HU1 (chain A)	5HU1 (chain B)
C1	-24.2807	-10.2254	-17.2824	-22.0899	-17.3930	-14.7416
C2	-24.0393	-24.6386	-26.9986	-16.5107	-25.7811	-17.7866

Table S8. Results of Molecular Docking of 45 Screened Flavonoids

AChE

	Docking score (kJmol^{-1})							
Comp.	1ACJ	1DX6	1EVE	1W6R	4EY6 (chain A)	4EY6 (chain B)	4EY7 (chain A)	4EY7 (chain B)
F1	-12.0618	-22.3781	-18.9030	-23.4092	-26.0704	-21.9408	-23.4116	-24.3408
F2	-21.3828	-21.1792	-23.2981	-27.0858	-21.9485	-21.5591	-28.7020	-30.9890
F3	-13.4475	-24.3904	-22.5304	-25.1471	-24.5858	-22.3901	-30.2081	-26.7821
F4	-16.0123	-22.8295	-22.2461	-19.7062	-20.6944	-19.0517	-21.4564	-21.3238
F5	-7.5262	-24.6627	-19.7850	-20.5093	-20.7438	-20.6685	-21.6841	-25.2464
F6	-13.5580	-18.7639	-21.9781	-18.6036	-20.3009	-14.1922	-20.9626	-21.0761
F7	-9.9819	-23.3978	-22.4062	-24.3032	-23.9333	-19.7709	-23.5900	-24.3024
F8	-19.5149	-22.8389	-22.8407	-20.9364	-21.8522	-22.6679	-22.6582	-22.8529
F9	-20.2663	-28.4602	-25.5345	-25.8010	-25.6296	-27.2649	-25.1678	-27.1133
F10	-16.5791	-22.6662	-18.0440	-24.8465	-22.1833	-20.5180	-22.5610	-23.4925
F11	-19.6910	-22.7552	-18.9415	-20.5700	-20.6336	-23.1171	-22.7930	-25.7063
F12	-28.2258	-28.4791	-26.3501	-30.4467	-29.3532	-30.9254	-28.9127	-33.8814
F13	-10.3653	-18.6836	-16.7264	-20.8186	-21.7743	-21.5369	-20.4117	-25.5742
F14	Not docked	-23.9202	-22.4849	-25.3507	-24.3460	-22.0860	-24.6864	-28.4700
F15	-22.4529	-24.6594	-23.8086	-19.3394	-21.0866	-22.9566	-33.5203	-31.8224
F16	Not docked	-25.2179	-19.6114	-22.4729	-21.2908	-16.7660	-25.4352	-27.0248
F17	-5.5811	-22.6225	-21.1004	-22.5060	-28.0683	-29.5975	-22.8341	-23.1114
F18	-14.6675	-26.8312	-16.1943	-22.5961	-22.5584	-26.6833	-27.2388	-25.8371
F19	-9.1813	-23.2421	-22.9078	-22.3891	-24.9791	-27.0724	-25.3394	-29.4205
F20	-21.5460	-20.8163	-21.6294	-21.4392	-23.7337	-20.7625	-25.8357	-25.1883
F21	Not docked	-14.7870	-17.7819	-17.5176	-24.6030	-20.9283	-20.9550	-21.2327
F22	-14.1209	-20.3342	-23.0535	-18.5286	-19.3338	-17.8781	-22.1666	-23.8686
F23	-11.4044	-20.8815	-22.5157	-19.0498	-19.5727	-18.4353	-20.6345	-24.8514
F24	-20.1935	-21.0835	-20.8666	-21.5331	-24.0959	-23.6559	-25.2652	-26.5880
F25	-21.5536	-32.0610	-27.5147	-28.7152	-25.8158	-27.9183	-37.6590	-35.6394
F26	-20.0899	-24.1971	-16.9469	-18.1873	-22.6949	-19.6880	-23.7688	-22.8605
F27	-14.1820	-16.9834	-13.2458	-20.7809	-21.1217	-18.2710	-26.5963	-24.0253
F28	-21.5076	-31.4750	-25.7109	-27.6128	-25.3963	-31.8587	-26.0085	-27.8597
F29	-12.9428	-23.6124	-27.1720	-21.2386	-26.3764	-29.2275	-22.1112	-24.0659
F30	-12.4444	-16.6759	-23.7233	-19.0557	-19.4550	-16.1588	-24.6189	-22.9723
F31	-11.2191	-16.8015	-21.4219	-17.6384	-20.7504	-16.1525	-23.7240	-25.7872
F32	-22.0626	-24.0253	-19.7194	-22.6002	-22.6114	-24.9575	-30.2555	-29.3262
F33	-22.7018	-22.9404	-22.6229	-22.7332	-22.9928	-22.1854	-28.3367	-23.9965
F34	-15.5600	-23.8956	-22.1222	-24.6781	-27.5752	-26.5423	-27.6069	-25.8094
F35	-17.1611	-17.8227	-13.8757	-19.3325	-23.2740	-22.7652	-29.0462	-24.6828
F36	-23.0233	-20.6052	-22.0908	-23.5264	-24.1917	-23.7474	-21.5136	-21.4094
F37	-20.5818	-22.4592	-21.8128	-22.9820	-21.0202	-22.7524	-23.3448	-22.4474
F38	-19.0606	-32.0050	-22.9780	-32.2649	-31.6249	-31.8352	-32.1923	-28.6235
F39	-20.4569	-27.0179	-25.8954	-25.3006	-25.0957	-25.1558	-27.8527	-28.1304
F40	-20.5126	-25.6165	-27.4122	-24.7137	-24.4960	-21.5195	-28.2155	-24.8449
F41	-17.6641	-19.3909	-17.7287	-21.6324	-21.0015	-21.9701	-20.9974	-20.7067
F42	-18.6271	-21.0632	-18.8899	-20.7085	-19.7733	-20.0073	-20.6404	-20.3391
F43	-12.2392	-19.8106	-23.9662	-20.3795	-22.1201	-20.9411	-19.5044	-21.3198
F44	-12.7998	-20.6217	-19.2991	-19.0739	-22.9853	-22.1737	-20.7577	-20.4096
F45	-18.8611	-30.3104	-24.4694	-23.1991	-24.4236	-23.3241	-35.9161	-31.6621

BACE-1

Comp.	Docking score (kJmol^{-1})					
	3VEU	4B78	5HU0 (chain A)	5HU0 (chain B)	5HU1 (chain A)	5HU1 (chain B)
F1	-24.0976	-14.9975	-19.0798	-17.2105	-24.9717	-23.1279
F2	-19.5067	-13.1095	-14.7868	-14.9229	-16.4678	-17.2022
F3	-23.2253	-14.3953	-17.2492	-17.8045	-19.6337	-19.1609
F4	-18.1798	-13.8693	-16.9733	-17.5194	-14.8097	-18.7405
F5	-23.2452	-13.3430	-21.0085	-15.0997	-13.9482	-14.9330
F6	-22.3548	-14.6395	-19.1106	-21.8683	-14.8273	-15.1312
F7	-27.6853	-15.1576	-19.0516	-18.3769	-17.0974	-17.1907
F8	-22.4403	-15.0131	-15.1116	-16.4599	-15.3285	-15.1951
F9	-21.3400	-15.9754	-18.5288	-17.8251	-20.3249	-19.4885
F10	-16.0028	-13.2684	-12.9418	-12.8389	-14.0145	-15.3733
F11	-21.6401	-10.9089	-16.0444	-17.8911	-16.1665	-13.4675
F12	-24.3245	-24.7768	-18.6106	-19.1233	-25.1448	-22.0106
F13	-18.6221	-12.3435	-14.8508	-13.7438	-14.4655	-17.5528
F14	-25.6240	-13.4531	-18.1305	-16.6662	-20.2198	-24.9800
F15	-19.4944	-13.8981	-15.4012	-14.4191	-18.2334	-18.4586
F16	-19.8738	-12.0599	-15.5066	-12.9914	-13.0521	-13.6387
F17	-21.3224	-16.0756	-16.8056	-16.6363	-17.0692	-20.8909
F18	-23.2231	-13.9112	-20.5961	-15.8662	-16.3414	-17.4564
F19	-23.5251	-14.8830	-18.6380	-17.9798	-19.2431	-18.8136
F20	-21.4360	-16.1004	-14.6125	-14.4733	-16.6811	-18.7916
F21	-17.9408	-15.5143	-14.4323	-15.5507	-14.3395	-15.0407
F22	-18.5452	-15.9043	-17.1959	-15.7601	-16.4728	-18.3509
F23	-18.8592	-15.3398	-17.5455	-17.1671	-16.2086	-17.9495
F24	-22.3912	-14.3622	-18.5844	-17.0652	-15.0872	-16.1928
F25	-24.6716	-18.5412	-17.0183	-17.4941	-21.2382	-21.9163
F26	-19.5872	-13.8563	-14.8729	-15.2658	-15.8633	-17.1390
F27	-16.9077	-11.1712	-12.9032	-12.8794	-12.7016	-16.0403
F28	-28.8694	-22.2429	-22.4020	-18.6305	-19.8570	-20.1542
F29	-22.6538	-16.9200	-19.5012	-19.5002	-15.5772	-20.6603
F30	-19.8772	-16.5934	-17.4529	-17.0162	-13.6939	-18.9678
F31	-20.4108	-15.9169	-17.7838	-16.7846	-14.4296	-19.6404
F32	-24.6975	-13.1931	-17.2961	-17.5663	-21.0621	-18.8726
F33	-24.6641	-13.2078	-18.0708	-16.2239	-20.5120	-22.2830
F34	-22.6318	-16.5918	-18.4368	-17.2444	-19.8521	-20.7125
F35	-16.0323	-12.7187	-15.1482	-15.6867	-13.2909	-16.7776
F36	-21.3753	-14.9042	-17.6353	-14.6895	-19.7802	-17.8277
F37	-21.8748	-13.6565	-17.3869	-15.8044	-16.6073	-15.0882
F38	-32.3704	-18.6677	-21.2405	-18.6529	-25.5348	-24.9926
F39	-24.7390	-20.9625	-19.8461	-19.4963	-18.5605	-24.0587
F40	-23.7950	-17.1070	-19.8752	-19.5461	-19.0494	-18.8044
F41	-25.1549	-14.5397	-16.9936	-18.1895	-15.5053	-15.2626
F42	-23.1142	-15.3050	-15.6572	-17.5268	-15.0859	-17.7446
F43	-23.0732	-18.1867	-18.4967	-18.9183	-17.0441	-20.2954
F44	-16.8179	-15.1796	-14.9778	-15.4666	-13.7183	-13.6423
F45	-23.1191	-16.2966	-17.1661	-17.7517	-23.0367	-24.6280

Table S9. Predicted pIC_{50} of screened substances against AChE and BACE-1

**Flavonoid**								
Compound	AChE	BACE-1	Compound	AChE	BACE-1	Compound	AChE	BACE-1
F1	4.74	6.12	F16	4.93	6.56	F31	5.00	5.87
F2	4.65	6.30	F17	4.99	5.31	F32	4.72	5.35
F3	4.74	6.22	F18	4.83	6.44	F33	4.72	5.76
F4	4.96	5.40	F19	4.99	6.44	F34	4.90	6.37
F5	4.80	6.47	F20	4.86	5.81	F35	4.57	4.73
F6	4.93	6.48	F21	4.60	5.91	F36	4.84	5.71
F7	4.97	6.13	F22	4.97	5.55	F37	4.84	6.11
F8	4.87	6.61	F23	4.96	5.94	F38	5.00	4.53
F9	4.86	6.77	F24	4.87	6.44	F39	4.76	4.96
F10	4.77	5.57	F25	4.72	5.58	F40	4.75	5.35
F11	4.90	6.31	F26	5.07	6.38	F41	5.09	5.47
F12	4.69	5.69	F27	4.55	4.85	F42	5.11	5.63
F13	4.87	6.16	F28	4.78	5.66	F43	4.97	5.42
F14	4.78	6.10	F29	4.96	5.26	F44	4.56	4.94
F15	4.68	6.23	F30	5.00	5.49	F45	4.70	5.15
**Curcumin**								
1	4.37	10.27						
2	4.24	9.13						

Table S10. Structures of 47 screened substances

Comp.	Structure	Comp.	Structure
**F1**	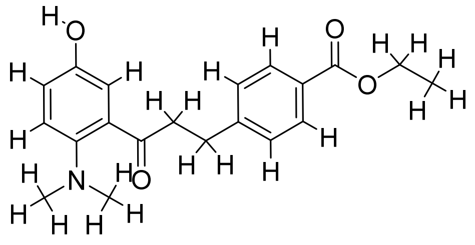	**F25**	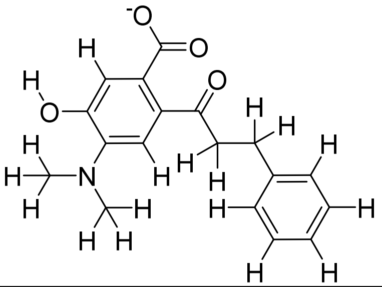
**F2**	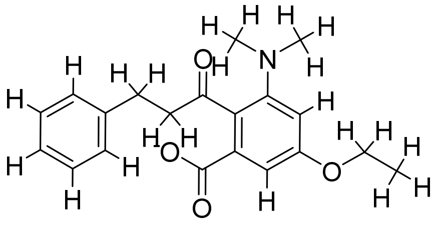	**F26**	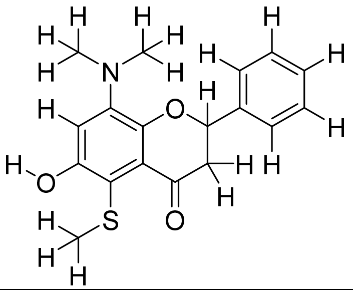
**F3**	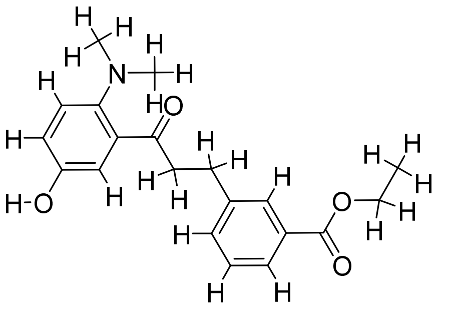	**F27**	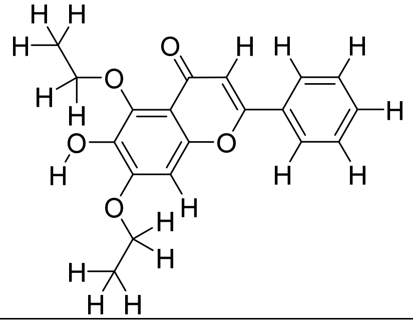
**F4**	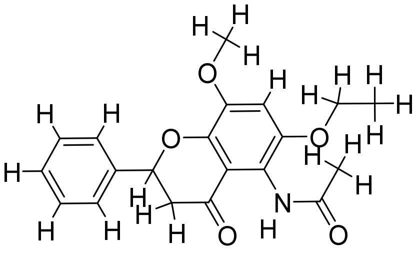	**F28**	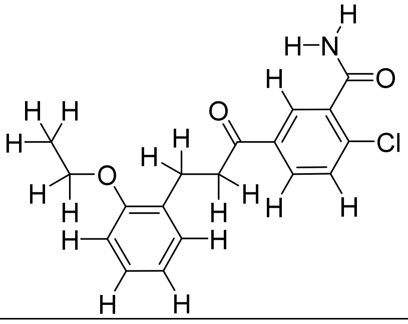
**F5**	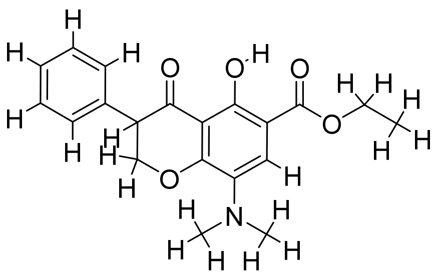	**F29**	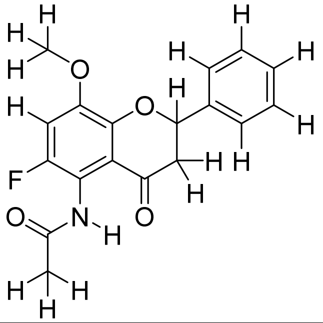

**F6**	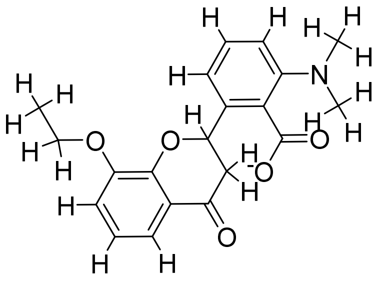	**F30**	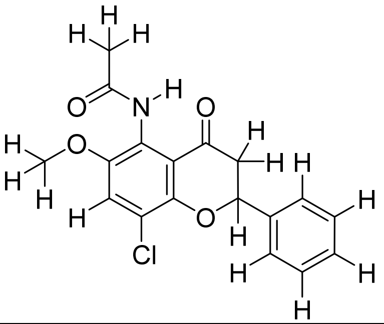
**F7**	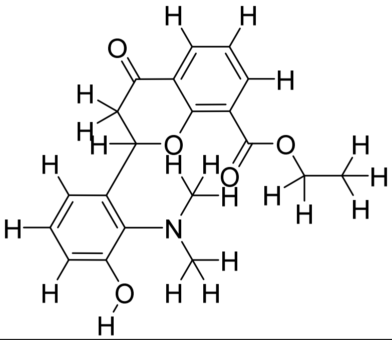	**F31**	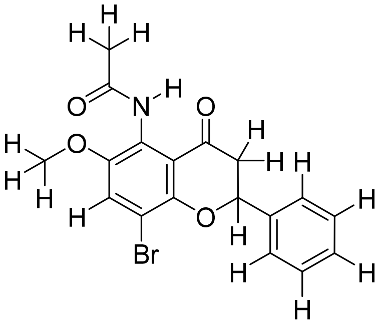
**F8**	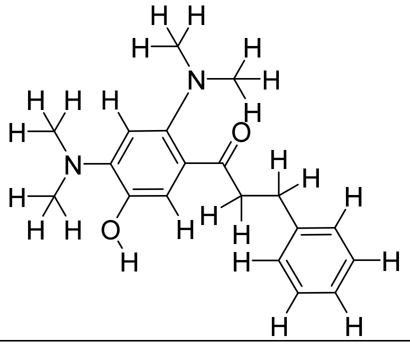	**F32**	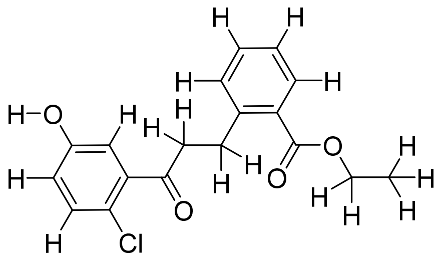
**F9**	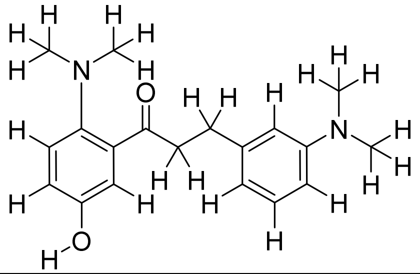	**F33**	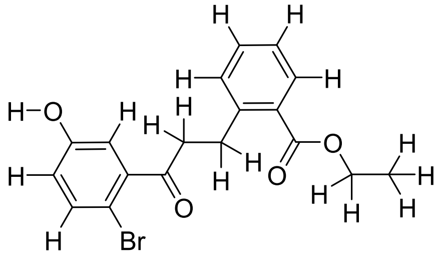
**F10**	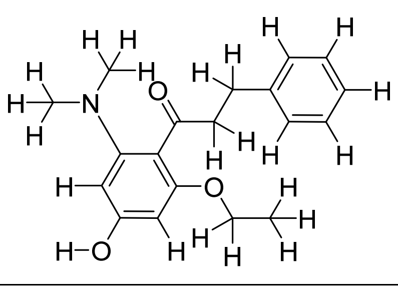	**F34**	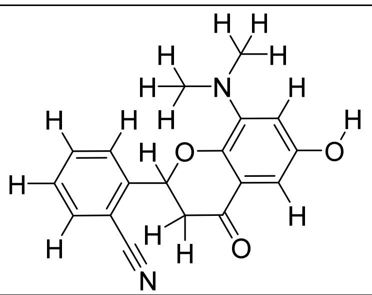

**F11**	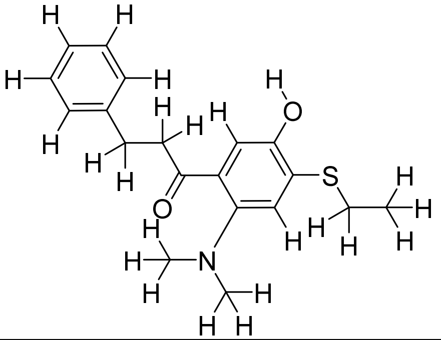	**F35**	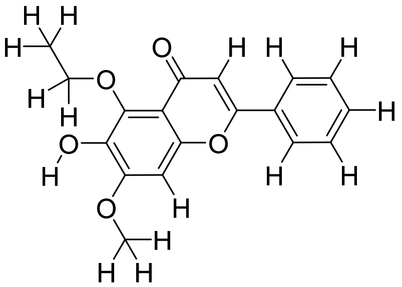
**F12**	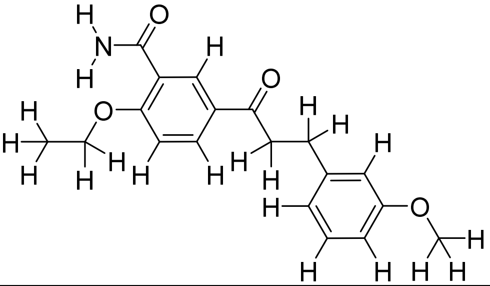	**F36**	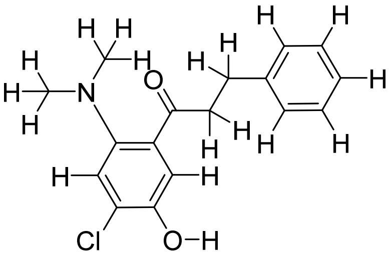
**F13**	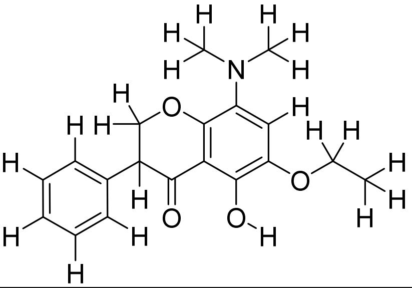	**F37**	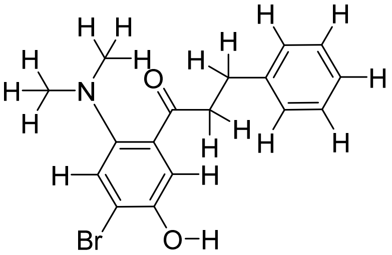
**F14**	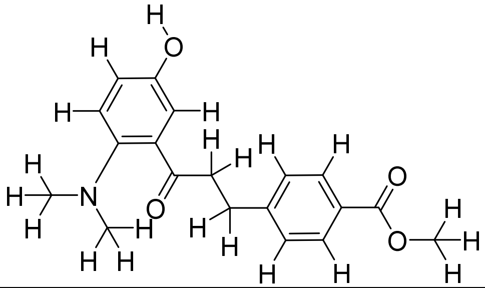	**F38**	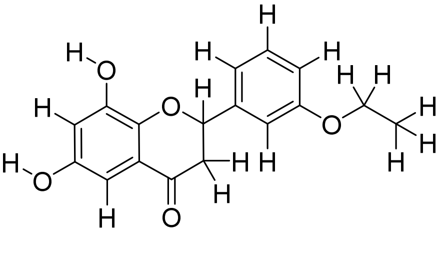
**F15**	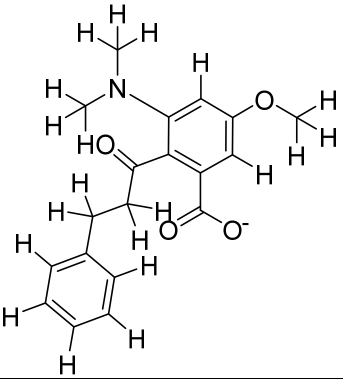	**F39**	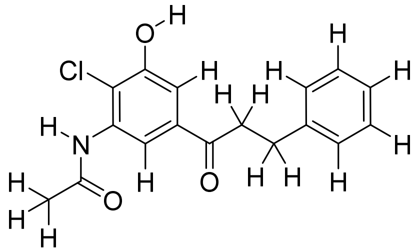

**F16**	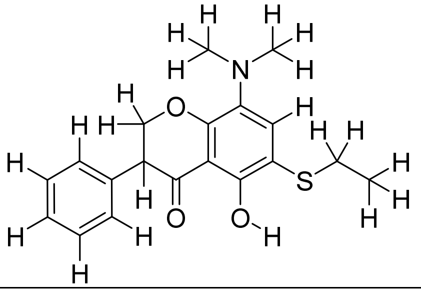	**F40**	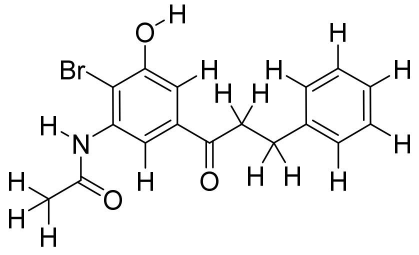
**F17**	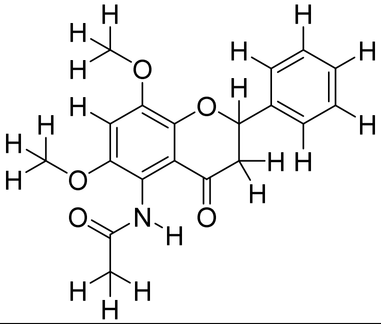	**F41**	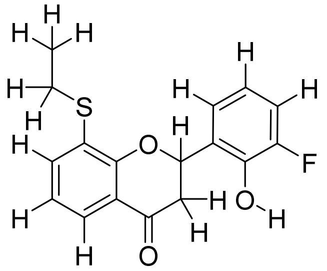
**F18**	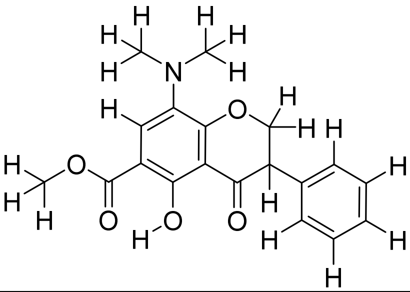	**F42**	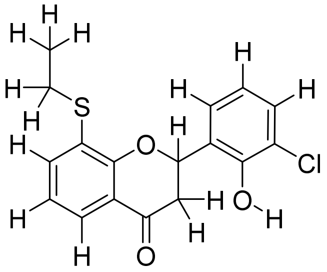
**F19**	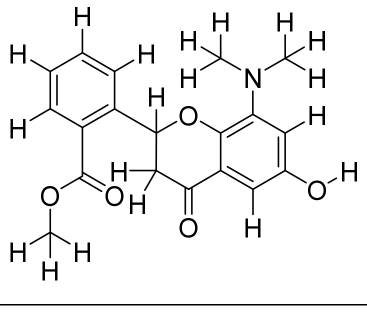	**F43**	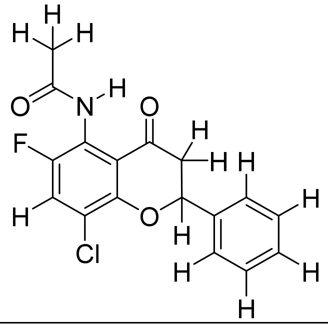
**F20**	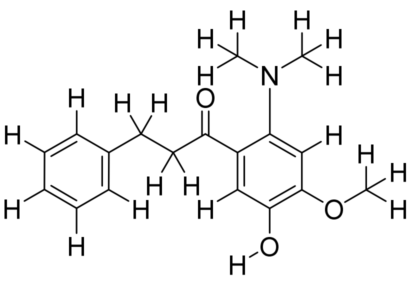	**F44**	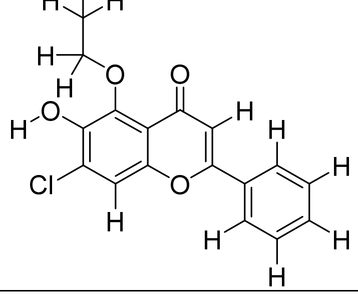

**F21**	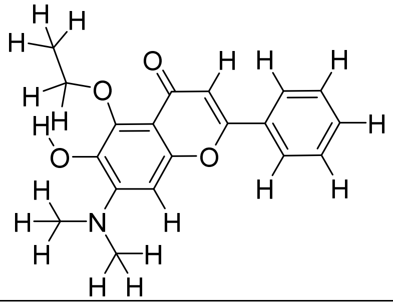	**F45**	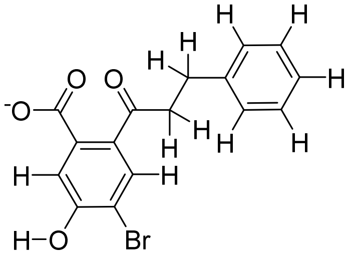
**F22**	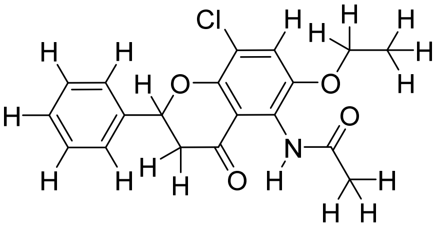	**C1**	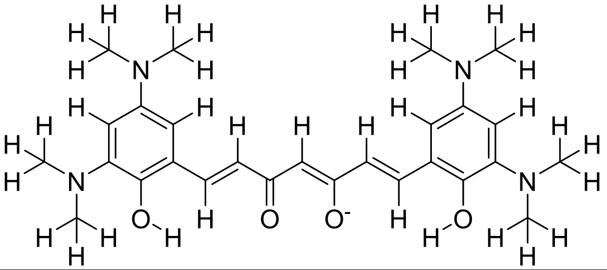
**F23**	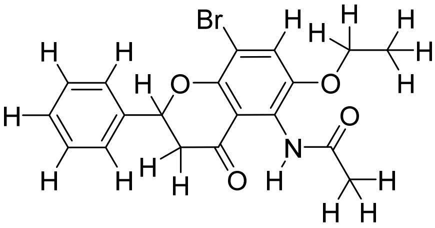	**C2**	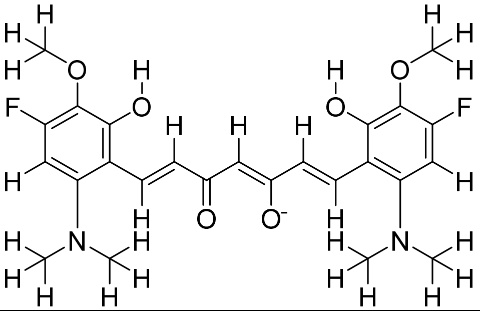
**F24**	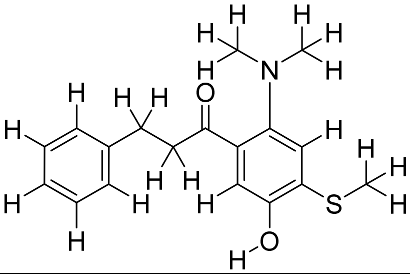		
